# Normal values for cardiovascular magnetic resonance in adults and children

**DOI:** 10.1186/s12968-015-0111-7

**Published:** 2015-04-18

**Authors:** Nadine Kawel-Boehm, Alicia Maceira, Emanuela R Valsangiacomo-Buechel, Jens Vogel-Claussen, Evrim B Turkbey, Rupert Williams, Sven Plein, Michael Tee, John Eng, David A Bluemke

**Affiliations:** Department of Radiology, Kantonsspital Graubuenden, Loestrasse 170, 7000 Chur, Switzerland; Cardiac Imaging Unit, Eresa Medical Center, C/Marqués de San Juan s/n, 46015 Valencia, Spain; Division of Paediatric Cardiology, University Children’s Hospital Zurich, Steinwiesstrasse 75, 8032 Zurich, Switzerland; Department of Diagnostic and Interventional Radiology, Hannover Medical School, OE 8220, Carl-Neuberg-Str 1, 30625 Hannover, Germany; Radiology and Imaging Sciences/ Clinical Image Processing Service, Clinical Center, NIH, 10 Center Drive, Bethesda, MD 20892 USA; The Rayne Institute, King’s College London, St Thomas’ Hospital, London, SE1 7EH UK; Multidisciplinary Cardiovascular Research Centre & Leeds Institute for Cardiovascular and Metabolic Medicine, LIGHT Laboratories, Clarendon Way, University of Leeds, Leeds, LS2 9JT UK; Radiology and Imaging Sciences, National Institute of Biomedical Imaging and Bioengineering, 10 Center Drive, Bethesda, MD 20892-1074 USA; Russell H. Morgan Department of Radiology and Radiological Science, Johns Hopkins University School of Medicine, 600 North Wolfe Street, Baltimore, MD 21287 USA

**Keywords:** Normal values, Reference values, Cardiovascular magnetic resonance

## Abstract

Morphological and functional parameters such as chamber size and function, aortic diameters and distensibility, flow and T1 and T2* relaxation time can be assessed and quantified by cardiovascular magnetic resonance (CMR). Knowledge of normal values for quantitative CMR is crucial to interpretation of results and to distinguish normal from disease. In this review, we present normal reference values for morphological and functional CMR parameters of the cardiovascular system based on the peer-reviewed literature and current CMR techniques and sequences.

**Electronic supplementary material:**

The online version of this article (doi:10.1186/s12968-015-0111-7) contains supplementary material, which is available to authorized users.

## Introduction

Quantitative cardiovascular magnetic resonance (CMR) is able to provide a wealth of information to help distinguish health from disease. In addition to defining chamber sizes and function, CMR can also determine regional function of the heart as well as tissue composition (myocardial T1 and T2* relaxation time). Advantages of quantitative evaluation are objective differentiation between pathology and normal conditions, grading of disease severity, monitoring changes under therapy and evaluating prognosis.

Knowledge of normal values is required to interpret the disease state. Thus, the aim of this review is to provide normal reference values for morphological and functional CMR parameters of the cardiovascular system based on a systematic review of the literature using current CMR techniques and sequences. Technical factors such as sequence parameters are relevant for CMR, and these factors are provided as in relationship to the normal values. In addition, factors related to post processing will affect the CMR analysis, and these factors are also described. When multiple peer-reviewed manuscripts are available for normal values, we describe the criteria used to select data for inclusion into this review. When feasible, we provide weighted means based on these literature values. Finally, demographic factors (e.g. age, gender, and ethnicity) may have an influence on normal values and are specified in the review.

## Statistical analysis

Results from multiple studies reporting normal values for the same CMR parameters were combined using a random effects meta-analysis model as implemented by the metan command [1]. This produced a weighted, pooled estimate of the population mean of the CMR parameters in the combined studies. Upper and lower limits were calculated as ±2SDp, where SD_p_ is the pooled standard deviation calculated from the standard deviations reported in each study [2]. Statistical analyses were performed with the Stata software package (version 13.1, StataCorp, College Station, TX).

## Left ventricular dimensions and functions in the adult

### CMR acquisition parameters

The primary method used to assess the left ventricle is steady state free precession (SSFP) technique at 1.5 Tesla. Steady-state free precession (SSFP) technique yields significantly improved blood-myocardium contrast compared to conventional fast gradient echo (FGRE). However, at 3 Tesla, fast gradient echo CMR may also be used. To date however, no studies have presented normal data at 3 Tesla. The derived cardiac volumes and ventricular mass are known to differ for SSFP and FGRE CMR, so that normal ranges are different for each method [3].

Publications presenting reference values of the left ventricle based on the SSFP technique are listed in Table 1.

**Table 1:** **References, normal LV function and dimensions, SSFP technique, axial imaging**

**First author, year**	**CMR technique**	**N, male: female**	**Age range (yrs)**
Alfakih, 2003 [3]	Short axis SSFP, papillary muscle included in LV mass	30:30	20-65
Hudsmith, 2005 [4]	Short axis SSFP, papillary muscle included in LV mass	63:45	21-68
Maceira, 2006 [5]	Short axis SSFP, papillary muscle included in LV mass	60:60	20-80

SSFP = steady-state free precession; LV = left ventricle; yrs = years.

### CMR analysis methods

*Papillary muscle mass* has been shown to significantly affect LV volumes and mass [6]. No uniformly accepted convention has been used for analyzing trabeculation and papillary muscle mass [7]. Papillary muscle mass has been noted to account for approximately 9% of total LV mass using FGRE technique [6]. Thus, tables of normal values should specify the status of the papillary muscles in the CMR analysis. Tables 2 and 3 provide normal values based on papillary muscle mass added to the remainder of the myocardial mass.

**Table 2:** **Left ventricular parameters, ages 20–80**

	**Men**			**Women**		
	**mean**_**p**_	**SD**_**p**_	Lower/ upper limits*	**mean**_**p**_	**SD**_**p**_	Lower/ upper limits*
EDV [ml]	160	27	106-214	132	23	86-178
EDV /BSA [ml/m^2^]	81	12	57-105	76	10	56-96
ESV [ml]	54	14	26-82	44	11	22-66
ESV/BSA [ml/m^2^]**	26	6	14-38	24	5	14-34
SV [ml]	108	18	72-144	87	15	57-117
SV/BSA [ml/m^2^]**	54	6	42-66	52	7	38-66
EF [%]	67	5	57-77	67	5	57-77
Mass [g]	134	21	92-176	98	21	56-140
Mass/BSA [g/m^2^]	67	9	49-85	61	10	41-81

LV papillary muscle mass *included as part of LV mass*. Pooled weighted mean values from references [3-5]. Mean_p_ = pooled weighted mean; SD_p_ = pooled standard deviation; * = calculated as mean_p_ ± 2*SD_p_; EDV = end-diastolic volume; ESV = end-systolic volume; SV = stroke volume; EF = ejection fraction; BSA = body surface area; SD = standard deviation; **from references [4,5] only.

**Table 3:** Left ventricular parameters, by age and gender [mean ± SD (lower, upper limits*)]

	**Men**		**Women**	
**Parameter**	**<60 years**	**≥60 years**	**<60 years**	**≥60 years**
EDV [ml]	161 ± 21 (119, 203)	148 ± 21 (106, 190)	132 ± 21 (90, 174)	120 ± 21 (78, 162)
EDV /BSA [ml/m^2^]	82 ± 9 (64, 100)	76 ± 9 (58, 94)	78 ± 8.7 (61, 95)	69 ± 8.7 (52, 86)
ESV [ml]	55 ± 11 (33, 77)	48 ± 11 (26, 70)	44 ± 9.5 (25, 63)	38 ± 9.5 (19, 57)
ESV/BSA [ml/m^2^]	28 ± 5.5 (17, 39)	25 ± 5.5 (14, 36)	26 ± 4.7 (17, 35)	22 ± 4.7 (13, 31)
SV [ml]	106 ± 14 (78, 134)	100 ± 14 (72, 128)	88 ± 14 (60, 116)	82 ± 14 (54, 110)
SV/BSA [ml/m^2^]	55 ± 6.1 (43, 67)	52 ± 6.1 (40, 64)	52 ± 6.2 (40, 64)	47.5 ± 6.2 (35, 60)
EF [%]	66 ± 4.5 (57, 75)	68 ± 4.5 (59, 77)	67 ± 4.6 (58, 76)	69 ± 4.6 (60, 78)
Mass [g]	147 ± 20 (107, 187)	145 ± 20 (105, 185)	106 ± 18 (70, 142)	110 ± 18 (74, 146)
Mass/BSA [g/m^2^]	74 ± 8.5 (57, 91)	73 ± 8.5 (56, 90)	62 ± 7.5 (47, 77)	63 ± 7.5 (48, 78)

LV papillary muscle mass *included as part of LV mass*. From reference [5].

* = calculated as mean ±2*SD; EDV = end-diastolic volume; ESV = end-systolic volume; SV = stroke volume; EF = ejection fraction; BSA = body surface area; SD = standard deviation.

**Figure 1:**
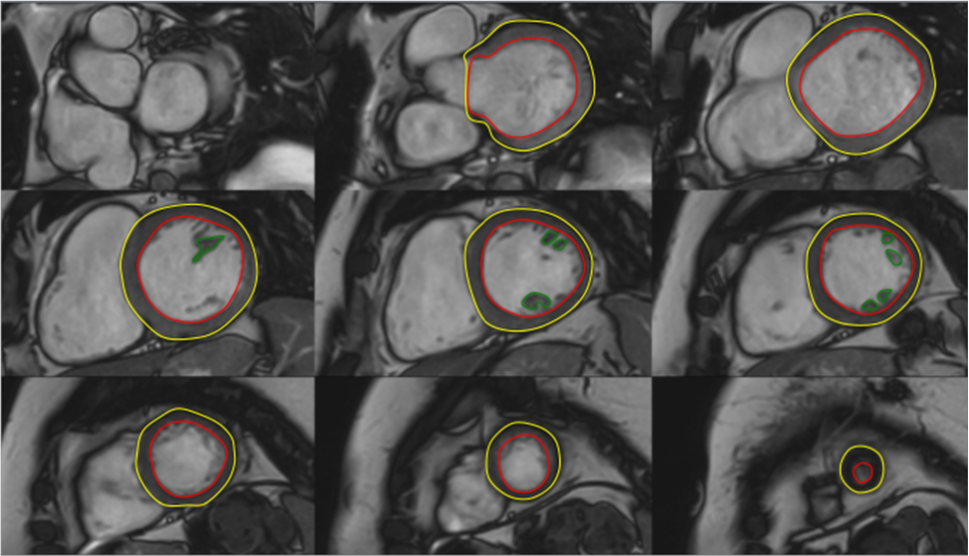
**LV contouring.** Note that LV papillary muscle mass has been isolated and added to left ventricular mass.

The majority of software approaches use a combination of semi-automated feature recognition combined with manual correction of contours. Short-axis images are most commonly analyzed on a per-slice bases by applying the Simpson’s method (“stack of disks”) [8]. An example of left ventricular contouring is shown in Figure 1.

### Demographic parameters

Gender has been demonstrated to have significant independent influence on ventricular volumes and mass. All absolute and normalized volumes decrease in relationship to age in adults [5] in a continuous manner. When considering younger (e.g. <65 years) versus older adults (≥65 years), most studies have shown significant differences in normal values for mass and volumes. For convenience, both average, and younger/ older normal values are given in the tables as available in the literature. An age-related normal value may be useful for patients who are at the upper or lower limits of the values in Tables 2 and 4.

**Table 4:** **Functional and geometric parameters of the normal left ventricle in the adult, from reference** [5]

	**Men**	**Women**
PFRE [ml/s]	527 ± 140 (253, 802)	477 ± 146 (190, 764)
PFRE /BSA [ml/m^2^]	270 ± 70 (134, 407)	279 ± 81 (121, 437)
PFRE/EDV [/s]	3.4 ± 0.71 (2.0, 4.8)	3.8 ± 0.83 (2.1, 5.4)
PFRA [ml/s]	373 ± 82 (212, 534)	283 ± 69 (149, 418)
PFRA/BSA [ml/m^2^]	193 ± 44 (107, 279)	168 ± 44 (82, 254)
PFRA/EDV [/s]	2.6 ± 0.57 (1.5, 3.7)	2.3 ± 0.49 (1.4, 3.3)
PFRE/PFRA	1.4 ± 0.34 (0.7, 2.8)	1.7 ± 0.29 (0.9, 3.1)
Septal AVPD [mm]	15 ± 3.6 (8, 22)	14 ± 3.2 (8, 21)
Septal AVPD /long length [%]	15 ± 2.9 (9, 21)	16 ± 3.5 (9, 23)
Lateral AVPD [mm]	18 ± 4.1 (9, 26)	17 ± 3.2 (11, 24)
Lateral AVPD /long length [%]	17 ± 3.2 (11, 23)	19 ± 3.1 (13, 24)
Sphericity index, diastole	0.35 ± 0.06 (0.22, 0.48)	0.4 ± 0.07 (0.27, 0.53)
Sphericity index, systole	0.20 ± 0.05 (0.10, 0.29)	0.23 ± 0.068 (0.09, 0.36)

Means ± standard deviation and (95% confidence intervals) are given.

BSA = body surface area; PFR = peak filling rate; E = early; A = active; AVPD = atrioventricular plane descent.

### Studies included in this review

Multiple studies have presented cohorts of normal individuals for determining normal dimensions of the left ventricle. For the purpose of this review, only cohorts of 30 or more normal subjects by gender using SSFP CMR have been included. Only data at 1.5T is available for normal subjects using SSFP short axis imaging. Inclusion criteria for the tables below also included a full description of the subject cohort (including the analysis methods used), age and gender of subjects. One study used SSFP radial imaging, and is not included in this review [9].

Multiple studies (not shown in the tables) have used FGRE technique at 1.5T [9-13]. While FGRE is currently used at 3T in some settings, the relevance of FGRE technique at 1.5T to that at 3T is not known.

**Figure 2:**
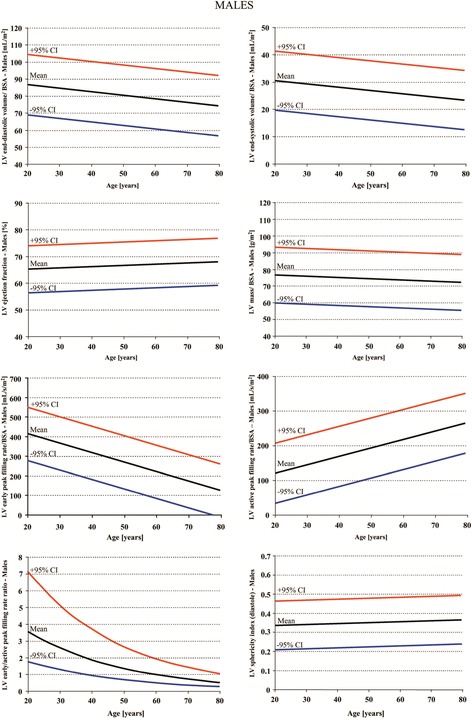
**Left ventricular volumes, mass and function in systole and diastole normalized to age and body surface area for males according to reference [** 5 **].**

**Figure 3:**
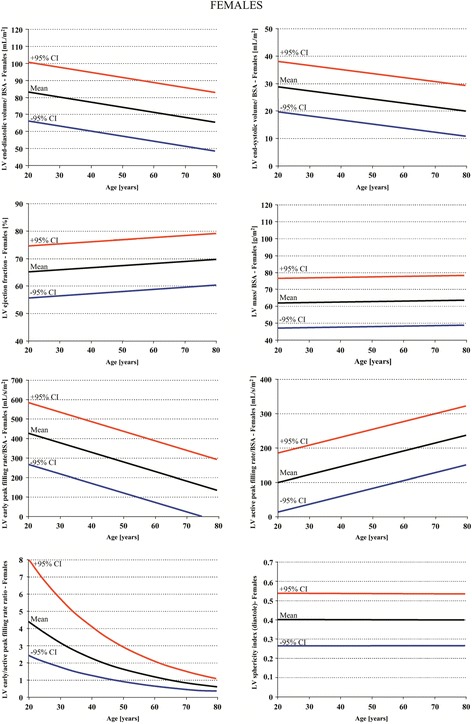
**Left ventricular volumes, mass and function in systole and diastole normalized to age and body surface area for females according to reference [** 5 **].**

Because slice FGRE acquisition parameters at 3T are different than at 1.5T, adaptation of 1.5T FGRE normal parameters to 3T FGRE imaging is not recommended. Information on ethnicity in relationship to LV parameters is not available for SSFP technique. Finally, the studies in Table 1 were all conducted in European centers. Normal values for left ventricular dimensions and functions according to these studies are presented in Tables 2, 3 and 4. Age dependent normal values for men and women are also presented in Figures 2 and 3.

### Additional LV function parameters

In addition to ejection fraction, Maceira et al. have provided additional functional parameters that may be useful in some settings [5]. For diastolic function, the derivative of the time/ volume filling curve expresses the peak filling rate (PFR). Both early (E) and active (A) filling rates are provided. In addition, longitudinal atrioventricular plane descent (AVPD) and sphericity index (volume observed/volume of sphere using long axis as diameter) at end diastole and end systole are given. These latter parameters are not routinely used for clinical diagnosis.

## Right ventricular dimensions and functions in the adult

### CMR acquisition parameters

For measurement of right ventricular volumes a stack of cine SSFP images acquired either horizontally or in short axis view can be used [7].

### CMR analysis methods

**Figure 4:**
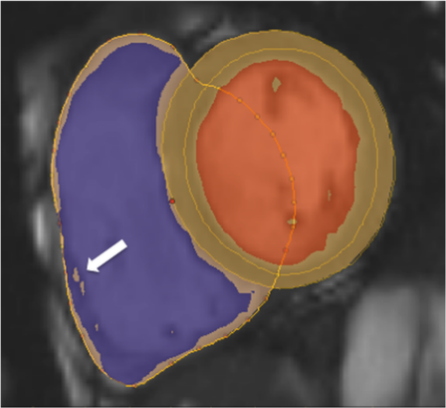
**Example of RV contouring using a semiautomatic software.** Note that papillary muscles were included in RV mass (arrow).

Similar to the left ventricle, analysis of the right ventricle is usually performed on a per slice basis by manual contouring of the endocardial and epicardial borders. Volumes are calculated based on the Simpson’s method [8]. The right ventricular volumes and mass are significantly affected by inclusion or exclusion of trabeculations and papillary muscles [14,15]. For manual contouring, inclusion of trabeculations and papillary muscles as part of the right ventricular volume will achieve higher reproducibility [7,14,15]. However, semiautomatic software is increasingly used for volumetric analysis, enabling automatic delineation of papillary muscles [16]. Therefore normal values for both methods are provided. An example of right ventricular contouring using a semiautomatic software is shown in Figure 4.

Detailed recommendations for right ventricular acquisitions and post processing have been published [7].

### Demographic parameters

BSA has been shown to have an independent influence on RV mass and volumes [16]. Absolute and normalized RV volumes are significantly larger in males compared to females [3,4,16]. Further, RV mass and volumes decrease with age [4,16].

### Studies included in this review

**Figure 5:**
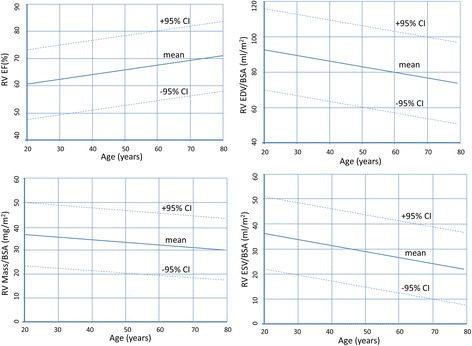
**Right ventricular volumes, mass and function for males by age decile.**

**Figure 6:**
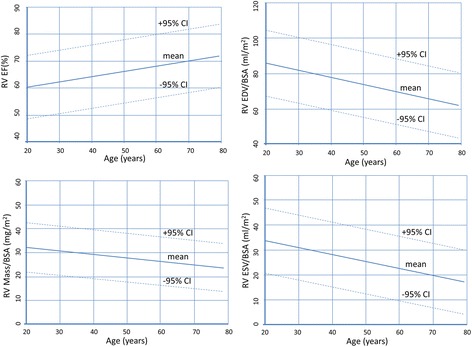
**Right ventricular volumes, mass and function for females by age decile.**

Criteria regarding study inclusion are identical compared to the left ventricle. Three studies based on SSFP imaging were included (Table 5). In two studies, trabeculations and papillary muscles were included as part of the right ventricular cavity [3,4], and pooled weighted mean values of the two studies are presented in Table 6. In the third study papillary muscles were considered part of the right ventricular mass [16]. Similar to the left ventricle, data is presented as a younger age (<60 years) and an older age group (≥60 years) (Table 7). Further, age dependent normal values for men and women are presented in Figures 5 and 6.

**Table 5:** **References, normal right ventricular function and dimensions, SSFP technique**

**First author, year**	**CMR technique**	**N, male: female**	**Age range (yrs)**
Alfakih, 2003 [3]	Short axis SSFP, papillary muscles and trabeculation included in RV volume	30:30	20-65
Hudsmith, 2005 [4]	Short axis SSFP, papillary muscles and trabeculation included in RV volume	63:45	21-68
Maceira, 2006 [16]	Short axis SSFP, papillary muscle included in RV mass	60:60	20-80

SSFP = steady-state free precession; RV = right ventricle; yrs = years.

**Table 6:** **Right ventricular parameters, ages 20–68**

	**Men**			**Women**		
**Parameter**	**mean**_**p**_	**SD**_**p**_	Lower/ upper limits*	**mean**_**p**_	**SD**_**p**_	Lower/ upper limits*
EDV [ml]	184	33	118-250	139	31	77-201
EDV /BSA [ml/m^2^]	91	15	61-121	80	16	48-112
ESV [ml]	79	19	41-117	54	15	24-84
ESV/BSA [ml/m^2^]**	39	10	19-59	32	10	12-52
SV [ml]	106	19	68-144	84	18	48-120
SV/BSA [ml/m^2^]**	57	8	41-73	53	9	35-71
EF [%]	62	5	52-72	61	5	51-71
Mass [g]**	41	8	25-57	35	7	21-49
Mass/BSA [g/m^2^]**	21	4	13-29	20	4	12-28

Right ventricular trabeculations and papillary muscle mass *included as part of right ventricular volume*.

Pooled weighted mean values from references [3,4].

mean_p_ = pooled weighted mean; SD_p_ = pooled standard deviation; * = calculated as mean_p_ ± 2*SD_p_; EDV = end-diastolic volume; ESV = end-systolic volume; SV = stroke volume; EF = ejection fraction; BSA = body surface area; SD = standard deviation; **from reference [4] only.

**Table 7:** Right ventricular parameters, by age and gender [mean ± SD (lower/upper limits*)]

	**Men**		**Women**	
**Parameter**	**<60 years**	**≥60 years**	**<60 years**	**≥60 years**
EDV [ml]	169 ± 25 (119, 219)	153 ± 25 (103, 203)	133 ± 22 (89, 177)	114 ± 22 (70, 158)
EDV /BSA [ml/m^2^]	87 ± 12 (63, 111)	77 ± 12 (53, 101)	78 ± 9 (60, 96)	66 ± 9 (48, 84)
ESV [ml]	62 ± 15 (32, 92)	48 ± 15 (18, 78)	49 ± 13 (23, 75)	35 ± 13 (9, 61)
ESV/BSA [ml/m^2^]	32 ± 7 (18, 46)	24 ± 7 (10, 38)	28 ± 7 (14, 42)	20 ± 7 (6, 34)
SV [ml]	107 ± 17 (73, 141)	105 ± 17 (71, 139)	85 ± 13 (59, 111)	80 ± 13 (54, 106)
SV/BSA [ml/m^2^]	55 ± 8 (39, 71)	53 ± 8 (37, 69)	50 ± 6 (38, 62)	46 ± 6 (34, 58)
EF [%]	64 ± 7 (50, 78)	69 ± 7 (55, 83)	64 ± 6 (52, 76)	70 ± 6 (58, 82)
Mass [g]	68 ± 14 (40, 96)	63 ± 14 (35, 91)	50 ± 11 (28, 72)	44 ± 11 (22, 66)
Mass/BSA [g/m^2^]	35 ± 7 (21, 49)	32 ± 7 (18, 46)	30 ± 5 (20, 40)	25 ± 5 (15, 35)

Right ventricular trabeculations and papillary muscle mass *included as part of right ventricular mass.* From reference [16].

EDV = end-diastolic volume; ESV = end-systolic volume; SV = stroke volume; EF = ejection fraction; BSA = body surface area; SD = standard deviation; * = calculated as mean ± 2*SD.

### Additional RV function parameters

Similar to the LV, Maceira et al. have provided additional functional parameters [16] (Table 8) that may have relevance to specific applications.

**Table 8:** **Functional and geometric parameters of the normal right ventricle in the adult, from reference** [16] **[mean ± SD (95% CI)]**

	**Men**	**Women**
PFRE [ml/s]	405 ± 137 (137, 674)	337 ± 117 (107, 567)
PFRE /BSA [ml/m^2^]	207 ± 70 (68, 345)	197 ± 68 (64, 330)
PFRE/EDV [/s]	2.4 ± 0.75 (1.0, 3.9)	2.7 ± 0.85 (1.0, 4.3)
PFRA [ml/s]	489 ± 175 (146, 833)	368 ± 153 (67, 668)
PFRA/BSA [ml/m^2^]	250 ± 94 (66, 434)	215 ± 89 (40, 390)
PFRA/EDV [/s]	3.1 ± 1.0 (1.0, 5.2)	2.9 ± 1.0 (0.9, 5.0)
PFRE/PFRA	0.8 ± 0.49 (−0.1, 1.8)	0.9 ± 0.46 (0.0, 1.8)
Septal AVPD [mm]	15 ± 4.1 (6, 23)	13 ± 3.0 (7, 19)
Septal AVPD /long length [%]	17 ± 4.5 (8, 26)	17 ± 3.9 (9, 25)
Lateral AVPD [mm]	22 ± 4.4 (13, 30)	21 ± 3.5 (14, 27)
Lateral AVPD /long length [%]	23 ± 4.1 (15, 31)	24 ± 4.0 (16, 32)

BSA = body surface area; PFR = peak filling rate; E = early; A = active; AVPD = atrioventricular plane descent; SD = standard deviation; CI = confidence interval.

## Left atrial dimensions and functions in the adult

### CMR acquisition parameters

There is limited consensus in the literature about how to measure left atrial volumes. Therefore depending on the method that is used, SSFP sequences in different views are required. The most common methods to measure left atrial volume are the modified Simpson’s method analogous to the left and right ventricle and the biplane area-length method. Dedicated 3D-modeling software has also been used [17]. For evaluation by applying the Simpson’s method, a stack of cine SSFP images either in the short axis, the horizontal long axis or transverse view is required. For 3-dimensional modeling a stack of short axis images has been used [17]. Evaluation by the biplane area-length method is based on a 2 and 4 chamber view [4].

Left atrial longitudinal and transverse diameters and area have been measured on 2, 3, and 4 chamber cine SSFP images [17].

### CMR analysis methods

Generally the left atrial appendage is included as part of the left atrial volume while the pulmonary veins are excluded [4,17,18].

The maximal left atrial volume is achieved during ventricular systole. Using cine images, the maximum volume can be defined as last image before opening of the mitral valve. Accordingly the minimal left atrial volume can be defined as first image after closure of the mitral valve [19].

### Demographic parameters

Body surface area (BSA) has been shown to have a significant independent influence on left atrial volume and most diameters [17]. Per Sievers et al. [20], age is not an independent predictor of left atrial maximal volume [17] nor diameter in normal individuals. Men have a larger maximal left atrial volume compared to women [4,17].

### Studies included in this review

**Figure 7:**
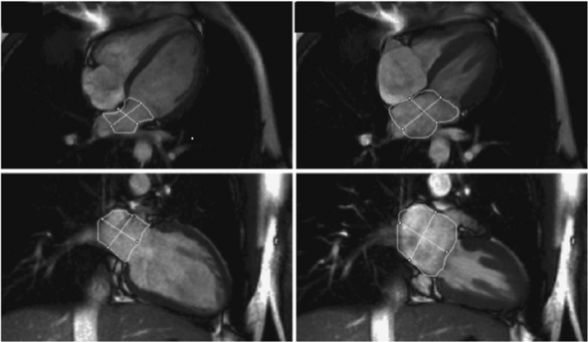
**Example of contouring for the biplane area-length method from reference [** 4 **].** The left atrial appendage was included in the atrial volume and the pulmonary veins were excluded.

**Figure 8:**
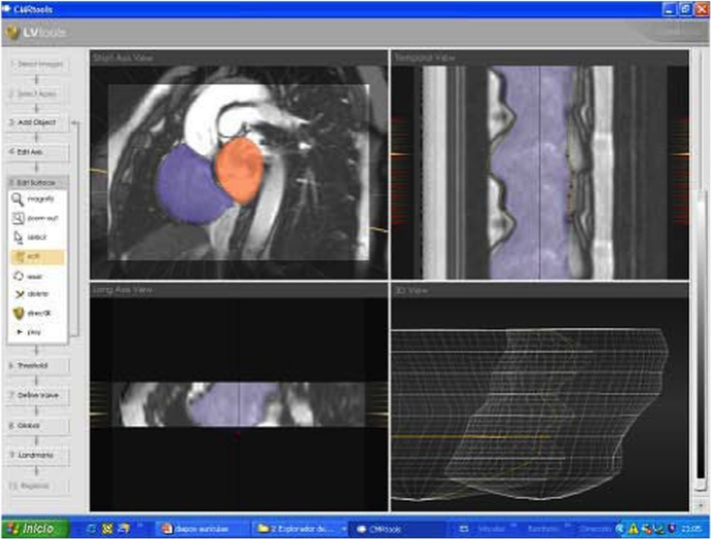
**Contouring of the left and right atrium using a 3D modeling method according to reference [** 17 **].**

**Figure 9:**
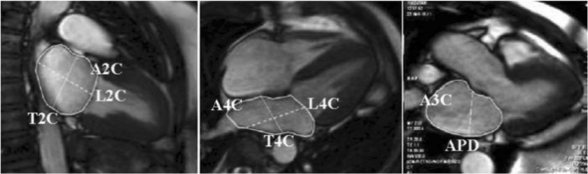
**Measurement of left atrial area (A2C, A4C, A3C), longitudinal (L2C, L4C), transverse (T2C, T4C) and anteroposterior (APD) diameters on the 2-, 4- and 3-chamber views according to reference [** 17 **].**

There are three publications for reference values of the left atrium (volume and/or diameter and/or area) based on SSFP imaging with a sufficient sample size [4,17,20] (Table 9). Hudsmith et al. [4] used the biplane area-length method (Figure 7), while Maceira et al. [17] used a 3D modeling technique (Figure 8). Since the results for left atrial maximal volume differ substantially between the two publications, probably based on the different methods, these data are presented separately (Tables 10 and 11, respectively). Maceira et al. provide reference values for maximum left atrial volume, longitudinal, transverse and anteroposterior diameters as well as area (Tables 11, 12 and 13; Figures 8 and 9) [17]. Hudsmith evaluated normal values of maximal and minimal left atrial volume and calculated left atrial ejection fraction and left atrial stroke volume (Table 10) [4]. Sievers et al. provide reference values for left atrial transverse diameters measured on the 2-, 3- and 4-chamber view at ventricular end-systole [20]. Maceira et al. provide both transverse and longitudinal diameters with different 3-chamber methodology than Sievers et al., thus only diameters of Maceira et al. are included (Table 13) [17,20].

**Table 9:** References, normal left atrial function and dimensions, SSFP technique

**First author, year**	**CMR technique**	**N, male: female**	**Age range (yrs)**
Sievers, 2005 [20]	2, 3 and 4 chamber SSFP; measurement of diameters	59:52	25-73
Hudsmith, 2005 [4]	2 and 4 chamber SSFP; biplane area-length method; atrial appendage included, pulmonary veins excluded	63:45	21-68
Maceira, 2010 [17]	Short axis, 2, 3 and 4 chamber SSFP; 3D modeling and measurement of area and diameters; atrial appendage included, pulmonary veins excluded (for volume analysis)	60:60	20-80

SSFP = steady-state free precession; yrs = years.

**Table 10:** **Left atrial volume and function in the adult for the SSFP technique based on the biplane area-length method according to reference** [4]

**Parameter**	**Men**			**Women**		
	**mean**	**SD**	Lower/ upper limits*	**mean**	**SD**	Lower/ upper limits*
Max. LA volume (ml)	103	30	43-163	89	21	47-131
Min. LA volume (ml)	46	14	18-74	41	11	19-63
EF (%)	55	13	29-81	53	9	35-71
SV (ml)	58	23	12-104	48	15	18-78

LA = left atrial; Max. = maximal; Min. = minimal; EF = ejection fraction; SV = stroke volume; SD = standard deviation; * = calculated as mean ± 2*SD.

**Table 11:** **Left atrial maximal volume in the adult for the SSFP technique based on 3D modeling methods, according to reference** [17]

**Parameter**	**Men**			**Women**		
	**mean**	**SD**	Lower/ upper limits*	**mean**	**SD**	Lower/ upper limits*
Max. LA volume (ml)	77	14.9	47-107	68	14.9	38-98
Max. LA volume/BSA (ml/m^2^)	39	6.7	26-52	40	6.7	27-53

LA = left atrial; Max. = maximal; BSA = body surface area; SD = standard deviation; * = calculated as mean ± 2*SD.

**Table 12:** **Left atrial maximal area in the adult for the SSFP technique, according to reference** [17]

**Parameter**	**Men**			**Women**		
	**mean**	**SD**	Lower/ upper limits*	**mean**	**SD**	Lower/ upper limits*
Area (cm^2^) 4ch	22	3.7	15-29	20	3.7	13-27
Area/BSA (cm^2^/ m^2^) 4ch	11	1.8	7-15	12	1.8	8-16
Area (cm^2^) 2ch	21	4.7	12-30	19	4.7	10-28
Area/BSA (cm^2^/ m^2^) 2ch	11	2.4	6-16	11	2.4	6-16
Area (cm^2^) 3ch	19	3.6	12-26	17	3.6	10-24
Area/BSA (cm^2^/ m^2^) 3ch	10	1.8	6-14	10	1.8	6-14

LA = left atrial; BSA = body surface area; SD = standard deviation; * = calculated as mean ± 2*SD; 4ch = 4-chamber view; 2ch = 2-chamber view; 3ch = 3-chamber view.

**Table 13:** **Left atrial diameter in the adult for the SSFP technique according to reference** [17]

**Parameter**	**Men**			**Women**		
	**mean**	**SD**	Lower/ upper limits*	**mean**	**SD**	Lower/ upper limits*
Longitudinal diameter (cm) 4ch	5.9	0.7	4.5-7.3	5.5	0.7	4.1-6.9
Longitudinal diameter/BSA (cm/m^2^) 4ch	3.0	0.4	2.2-3.8	3.2	0.4	2.4-4.0
Transverse diameter (cm) 4ch	4.1	0.5	3.1-5.1	4.1	0.5	3.1-5.1
Transverse diameter/BSA (cm/m^2^) 4ch	2.1	0.3	1.5-2.7	2.4	0.3	1.8-3.0
Longitudinal diameter (cm) 2ch	5.0	0.7	3.6-6.4	4.6	0.7	3.2-6.0
Longitudinal diameter/BSA (cm/m^2^) 2ch	2.5	0.4	1.7-3.3	2.7	0.4	1.9-3.5
Transverse diameter (cm) 2ch	4.6	0.5	3.6-5.6	4.4	0.5	3.4-5.4
Transverse diameter/BSA (cm/m^2^) 2ch	2.3	0.2	1.9-2.7	2.6	0.2	2.2-3.0
AP diameter (cm) 3ch	3.3	0.5	2.3-4.3	3.1	0.5	2.1-4.1
AP diameter/BSA (cm/m^2^) 3ch	1.7	0.3	1.1-2.3	1.8	0.3	1.2-2.4

BSA = body surface area; SD = standard deviation; * = calculated as mean ± 2*SD; 4ch = 4-chamber view; 2ch = 2-chamber view; 3ch = 3-chamber view; AP = anteroposterior.

## Right atrial dimensions and functions in the adult

### CMR acquisition parameters

There is no consensus in the literature regarding acquisition and measurement method for the right atrium. Published methods for right atrial volume include the modified Simpson’s method, the biplane area-length method and 3D-modeling [21,22]. Comparing the Simpsons method and the biplane area length method results in different values for right atrial volume [21]. For Simpson’s method and 3D modeling, a stack of cine SSFP images in the short axis view are analyzed. For the biplane area-length method, a 4-chamber view and/or a right ventricular 2-chamber view are evaluated.

### CMR analysis methods

Generally the right atrial appendage is included in the right atrial volume while the inferior and superior vena cava are excluded [21,22].

The maximal right atrial volume is achieved during ventricular systole and can be defined as last cine image before opening of the tricuspid valve. The minimal left atrial volume can be defined as first cine image after closure of the tricuspid valve.

### Demographic parameters

Maceira et al. demonstrated a significant independent influence of BSA on most RA parameters [22]. There was no influence of age on atrial parameters and no influence of gender on atrial volumes [21,22].

### Studies included in this review

There are two publications of reference values for the right atrium (volume and/or diameter) based on SSFP imaging with a sufficient sample size [21,22] (Table 14). For evaluation of volume, Maceira et al. [22] used a 3D modeling technique (Figure 8) while Sievers et al. [21] applied the Simpsons and the biplane area-length methods, respectively. Due to different methodology, no pooled mean values are provided. Normal values for right atrial volume and function, diameter and area are presented in Tables 15, 16, 17, 18 and 19.

**Table 14:** **References, normal RA function and dimensions, SSFP technique**

**First author, year**	**CMR technique**	**N, male: female**	**Age range (yrs)**
Sievers, 2007 [21]	Short axis SSFP and 4 chamber SSFP; Simpsons method and biplane area length method; atrial appendage included, pulmonary veins excluded	38:32	25-73
Maceira, 2013 [22]	Short axis SSFP; 3D modeling; atrial appendage included, pulmonary veins excluded	60:60	20-80

SSFP = steady-state free precession; yrs = years.

**Table 15:** **Right atrial volume and function in the adult for the SSFP technique based on the Simpson’s method according to reference** [21]

**Parameter**	**mean**	**SD**	Lower/ upper limits*
Max. RA volume (ml)	101	30	41-161
Max. RA volume/BSA (ml/ m^2^)	53	16	21-85
Min. RA volume (ml)	50	19	12-88
Min. RA volume/BSA (ml/m^2^)	27	10	7-47
SV (ml)	50	16	18-82
SV/BSA (ml/m^2^)	26	9	8-44
EF (%)	47	8	31-63

RA = right atrial; Max. = maximal; Min. = minimal; SV = stroke volume; EF = ejection fraction; SD = standard deviation; BSA = body surface area; * = calculated as mean ± 2*SD; since no influence of gender was demonstrated, gender specific values are not presented.

**Table 16:** **Right atrial volume and function in the adult for the SSFP technique based on the biplane-area-length method according to reference** [21]

**Parameter**	**mean**	**SD**	Lower/ upper limits*
Max. RA volume (ml)	103	33	37-169
Max. RA volume/BSA (ml/ m^2^)	54	18	18-90
Min. RA volume (ml)	51	20	11-91
Min. RA volume/BSA (ml/m^2^)	27	11	5-49
SV (ml)	52	17	18-86
SV/BSA (ml/m^2^)	27	9	9-45
EF (%)	51	9	33-69

RA = left atrial; Max. = maximal; Min. = minimal; SV = stroke volume; EF = ejection fraction; SD = standard deviation; BSA = body surface area; * = calculated as mean ± 2*SD; since no influence of gender was demonstrated, gender specific values are not presented.

**Table 17:** **Right atrial volume in the adult for the SSFP technique based a 3D modeling technique according to reference** [22]

**Parameter**	**mean**	**SD**	Lower/ upper limits*
Max. RA volume (ml)	100	20	60-140
Max. RA volume/BSA (ml/ m^2^)	54	10	34-74

RA = left atrial; Max. = maximal; * = calculated as mean ± 2*SD; BSA = body surface area; since no influence of gender was demonstrated, gender specific values are not presented.

**Table 18:** **Right atrial maximal area in the adult for the SSFP technique according to reference** [22]

**Parameter**	**mean**	**SD**	Lower/ upper limits*
Area (cm^2^) 4ch	22	3.8	14-30
Area/BSA (cm^2^/ m^2^) 4ch	12	1.8	8-16
Area (cm^2^) 2ch	22	3.95	14-30
Area/BSA (cm^2^/ m^2^) 2ch	12	2.27	7-17

BSA = body surface area; * = calculated as mean ± 2*SD; since no influence of gender was demonstrated, gender specific values are not presented.

**Table 19:** **Right atrial diameter in the adult for the SSFP technique according to reference** [22]

**Parameter**	**mean**	**SD**	Lower/ upper limits*
Longitudinal diameter (cm) 4ch	5.5	0.58	4.3-6.7
Longitudinal diameter/BSA (cm/m^2^) 4ch	3.0	0.32	2.4-3.6
Transverse diameter (cm) 4ch	4.7	0.55	3.6-5.8
Transverse diameter/BSA (cm/m^2^) 4ch	2.6	0.3	2.0-3.2
Longitudinal diameter (cm) 2ch	5.4	0.5	4.4-6.4
Longitudinal diameter/BSA (cm/m^2^) 2ch	2.9	0.3	2.3-3.5
Transverse diameter (cm) 2ch	4.3	0.7	2.9-5.7
Transverse diameter/BSA (cm/m^2^) 2ch	2.4	0.4	1.6-3.2

BSA = body surface area; SD = standard deviation; * = calculated as mean ± 2*SD; 4ch = 4-chamber view; 2ch = 2-chamber view; since no influence of gender was demonstrated, gender specific values are not presented.

## Left and right ventricular dimensions and function in children

The presentation of normal values in children is different than in the adult population due to continuous changes in body weight and height as a function of age. These changes may also be asymmetrical. Normal data in children is frequently presented in percentiles and/or z-scores. While the use of percentiles is a daily routine for the paediatric radiologist, the use of percentile data might be unfamiliar to the general radiologist. Therefore in the current review, normal values are presented as mean ± standard deviation as well as in percentiles.

### Demographic parameters

A linear correlation between ventricular volumes and BSA in children has been reported. Ventricular volumes also vary by gender [23-25]. Ejection fraction remains constant during somatic growth and does not appear to be gender specific [23-25]. Gender differences are more marked in older children, indicating that gender is more important after puberty and in adulthood.

### Studies included in this review

Normal values published in studies based on older gradient echo sequences are not comparable to current SSFP techniques [26,27]. Literature values for normative SSFP values have been proposed by three different groups acquired with slightly different methods [23-25] (Table 20). A good agreement between the three studies regarding the dimensions for older children has been demonstrated [25].

**Table 20:** **References, normal left and right ventricular dimensions in children**

**First author, year**	**CMR technique**	**N, male: female**	**Age range (yrs)**
Robbers-Visser, 2009 [23]	Short axis SSFP, papillary muscle/ trabeculation excluded from volumes	30:30*	8-17
Buechel, 2009 [25]	Short axis SSFP, papillary muscle/trabeculation included in volumes, separate analysis of papillary muscle mass	23:27**	7 mo – 18
Sarikouch, 2010 [24]	Axial SSFP (in 29 children additional short axis stack), papillary muscle/ trabeculation excluded from volumes	Total: 55:59* Percentiles: 51:48	Total: 4–20 Percentiles: 8-20

SSFP = steady-state free precession; yrs = years; mo = month; * = none of the subjects was sedated; ** = 13 subjects were sedated.

In the studies of Robbers-Visser et al. and Sarikouch et al., normal values for older children of 8–17 years and 4–20 years, respectively, are presented [23,24], Buechel et al. also include younger children starting with an age of 7 months. In the studies of Robbers-Visser et al. and Sarikouch et al. papillary muscle mass was included as part of LV mass, while Buchel et al. included papillary muscles in the LV cavity and provide separate values for papillary muscle mass. Robbers-Visser et al. and Sarikouch et al. present normal values as mean ± SD for all female and male children and for children of different age groups and also as percentiles. In the study by Buechel et al. data is presented in percentiles only.

**Figure 10:**
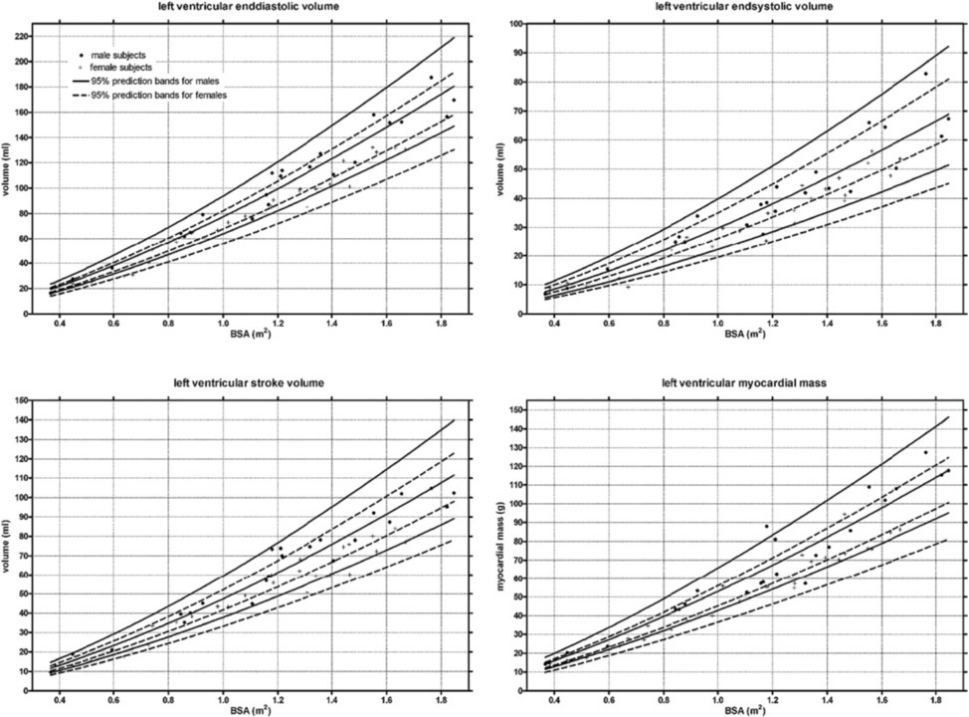
**Percentiles for left ventricular parameters in children according to reference [** 25 **].**

**Figure 11:**
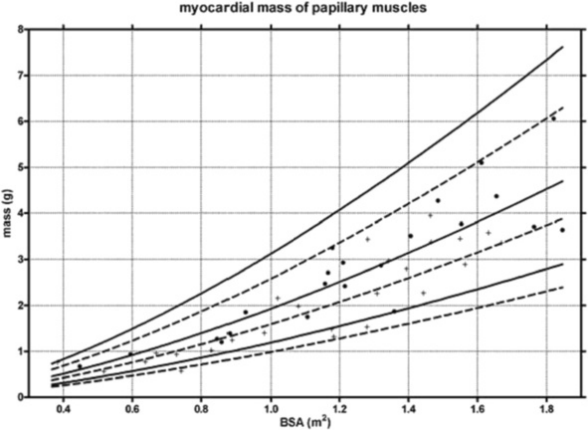
**Percentiles for left ventricular papillary muscle mass in children according to reference [** 25 **].**

**Figure 12:**
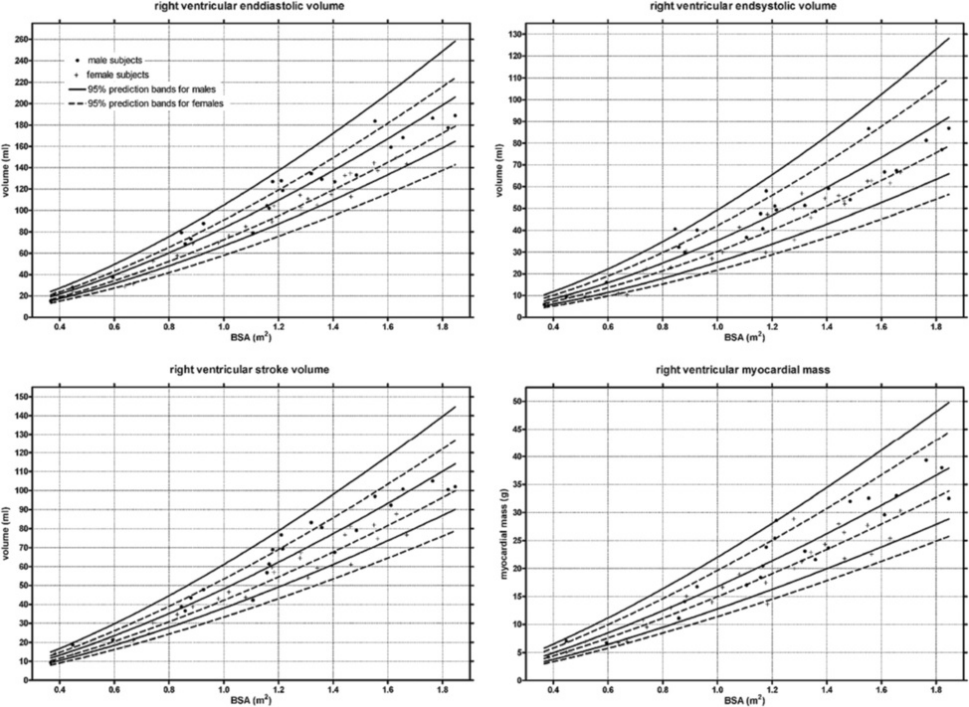
**Percentiles for right ventricular parameters in children according to reference [** 25 **].**

Due to similar study design and age range, Tables 21 and 22 show pooled mean values for male and female children calculated based on mean values presented by Robbers-Visser et al. and Sarikouch et al. Figures 10,11,12 show normal data in percentiles originally published by Buechel et al.

**Table 21:** **Left ventricular parameters in children, ages 8–17**

**Parameter**	**Male**			**Female**		
	**mean**_**p**_	**SD**_**p**_	Lower/ upper limits*	**mean**_**p**_	**SD**_**p**_	Lower/ upper limits*
EDV/BSA [ml/m^2^]	80	12	56-104	75	10	55-95
ESV/BSA [ml/m^2^]	28	6	16-40	25	5	15-35
SV/BSA [ml/m^2^]**	54	9	36-72	50	8	34-66
EF [%]**	66	5	56-76	63	6	51-75
CI [l/min/ m^2^]**	4.4	0.85	2.7-6.1	3.9	0.62	2.7-5.1
Mass/BSA [g/m^2^]	62	12	38-86	53	9	35-71

Left ventricular papillary muscle mass included as part of left ventricular mass. Pooled weighted mean values from references [23,24].

mean_p_ = pooled weighted mean; SD_p_ = pooled standard deviation; * = calculated as mean_p_ ± 2*SD_p_; EDV = end-diastolic volume; ESV = end-systolic volume; BSA = body surface area; SV = stroke volume; EF = ejection fraction; CI = cardiac index; SD = standard deviation; ** = from reference [24] (8–15 years) only.

**Table 22:** **Right ventricular parameters in children, ages 8–17**

**Parameter**	**Male**			**Female**		
	**mean**_**p**_	**SD**_**p**_	Lower/ upper limits*	**mean**_**p**_	**SD**_**p**_	Lower/ upper limits*
EDV/BSA [ml/m^2^]	84	12	60-108	76	9	58-94
ESV/BSA [ml/m^2^]	32	7	18-46	27	5	17-37
SV/BSA [ml/m^2^]**	52	8	36-68	49	7	35-63
EF [%]**	62	4	54-70	63	4	55-71
Mass/BSA [g/m^2^]	21	5	11-31	18	4	10-26

Right ventricular trabeculation included as part of right ventricular mass. Pooled weighted mean values from references [23,24].

mean_p_ = pooled weighted mean; SD_p_ = pooled standard deviation; * = calculated as mean_p_ ± 2*SD_p_ EDV = end-diastolic volume; ESV = end-systolic volume; BSA = body surface area; SV = stroke volume; EF = ejection fraction; SD = standard deviation; ** = from reference [24] only.

Z-values can be calculated as z-value = (measurement – expected mean)/SD by using the values presented in Tables 21 and 22.

## Left and right atrial dimensions and function in children

### CMR acquisition parameters

Left and right atrial dimensions and function were evaluated using SSFP technique in a single publication [18], (Table 23). Measurements were obtained on a stack of transverse cine SSFP images with a slice thickness between 5 and 6 mm without interslice gap [18].

**Table 23:** **Reference, normal left and right atrial dimensions in children**

**First author, year**	**CMR technique**	**N, male: female**	**Age range (yrs)**
Sarikouch, 2011 [18]	Axial SSFP; pulmonary veins, SVC, IVC and coronary sinus excluded, atrial appendages included from/in the left and right atrial volume, respectively	56:59	4.4-20.3

SSFP = steady-state free precession; SVC = superior vena cava; IVC inferior vena cava; yrs = years.

### CMR analysis methods

In that study, the pulmonary veins, the superior and inferior vena cava and the coronary sinus were excluded from the left and right atrial volume, respectively, while the atrial appendages were included in the volume of the respective atrium. The maximal atrial volume was measured at ventricular end-systole and the minimal atrial volume at ventricular end-diastole.

### Demographic parameters

Left and right atrial volumes show an increase with age with a plateau after the age of 14 for girls only. Absolute and indexed volumes have been shown to be significantly greater for boys compared to girls (except for the indexed maximal volumes for both atria) [18].

### Studies included in this review

Sarikouch et al. evaluated atrial parameters of 115 healthy children (Table 23) [18] using SSFP imaging. Since the standard deviation is large for each parameter, lower and upper limits were not calculated (Tables 24 and 25). Theoretically calculation of lower limits by mean – (2*SD) would result in negative lower limits for certain parameters.

**Table 24:** **Left atrial parameters in children, ages 4–20 according to reference** [18]

	**Male**		**Female**	
**Parameter**	**mean**	**SD**	**mean**	**SD**
Vol. max. [ml]	71	30	59	20
Vol. max./BSA [ml/m^2^]	47	10	44	9
Vol. min. [ml]	33	15	26	9
Vol. min./BSA [ml/m^2^]	22	5	19	4
SV [ml]	38	16	33	12
EF [%]	54	6	56	6

Vol. max. = maximal atrial volume; Vol. min. = minimal atrial volume; BSA = body surface area; SV = stroke volume; EF = ejection fraction; SD = standard deviation.

**Table 25:** **Right atrial parameters in children, ages 4–20 according to reference** [18]

	**Male**		**Female**	
**Parameter**	**mean**	**SD**	**mean**	**SD**
Vol. max. [ml]	89	43	71	25
Vol. max./BSA [ml/m^2^]	58	16	53	12
Vol. min. [ml]	42	21	31	13
Vol. min./BSA [ml/m^2^]	27	8	23	6
SV [ml]	47	24	40	15
EF [%]	53	7	56	8

Vol. max. = maximal atrial volume; Vol. min. = minimal atrial volume; BSA = body surface area; SV = stroke volume; EF = ejection fraction; SD = standard deviation.

## Normal left ventricular myocardial thickness

### CMR acquisition parameters

Normal values of left ventricular myocardial thickness (LVMT) have been shown to vary by type of pulse sequence (FGRE versus SSFP) [3,28]. For the purposes of this review, only SSFP normal values are shown.

### CMR analysis methods

Measures of LVMT vary by the plane of acquisition (short axis versus long axis) [29]. Measurements obtained on long axis images at the basal and mid-cavity level have been shown to be significantly greater compared to measurements on corresponding short axis images, whereas measurements obtained at the apical level of long axis images are significantly lower compared to short axis images. In recent publications, papillary muscles and trabeculations were excluded from measurements of left ventricular myocardial thickness [29,30].

### Demographic parameters

LVMT is greater in men than women [29,30]. There are also small differences in LVMT in relationship to ethnicity and body size, but these variations are not likely to have clinical significance [29]. Regarding age, one study of 120 healthy volunteers age 20–80 years reported an increase in myocardial thickness with age—starting after the fourth decade [30]. In the study by Kawel el al. of 300 normal individuals without hypertension, smoking history or diabetes, there was no statistically significant difference in LVMT with age [29].

### Studies included in this review

There are two publications of a systematic analysis of left ventricular myocardial thickness based on SSFP imaging at 1.5T [29,30]. In the study by Dawson et al., measurements were obtained on short axis images only. Kawel et al. published normal values of LVMT for long and short axis imaging (Tables 26, 27 and 28).

**Table 26:** **References, normal left ventricular myocardial thickness in adults**

**First author, year**	**CMR technique**	**N, male: female**	**Age range (yrs)**
Dawson, 2011 [30]	Short axis SSFP, papillary muscle/ trabeculation excluded from LVMT	60:60	20-80
Kawel, 2012 [29]	Short and long axis SSFP, papillary muscle/ trabeculation excluded from LVMT	131:169	54-91

SSFP = steady-state free precession; yrs = years; LVMT = left ventricular myocardial thickness.

**Table 27:** **Normal left ventricular myocardial thickness in mm measured on long axis images for men and women according to** [29]

		**Men**			**Women**		
**Level**	**Region**	**mean**	**SD**	Lower/upper limits*	**mean**	**SD**	Lower/upper limits*
basal	anterior	8.2	1.3	5.6-10.8	7.0	1.1	4.8-9.2
	inferior	8.2	1.3	5.6-10.8	6.7	1.1	4.5-8.9
	septal	9.1	1.3	6.5-11.7	7.3	1.1	5.1-9.5
	lateral	7.6	1.3	5.0-10.2	6.0	1.1	3.8-8.2
	**mean**	8.3	1.0	6.3-10.3	6.8	0.9	5.0-8.6
mid-cavity	anterior	6.0	1.3	3.4-8.6	4.9	1.1	2.7-7.1
	inferior	7.7	1.3	5.1-10.3	6.5	1.1	4.3-8.7
	septal	8.3	1.3	5.7-10.9	6.8	1.1	4.6-9.0
	lateral	6.6	1.3	4.0-9.2	5.3	1.1	3.1-7.5
	**mean**	7.2	1.0	5.2-9.2	5.9	0.9	4.1-7.7
apical	anterior	5.1	1.3	2.5-7.7	4.2	1.1	2.0-6.4
	inferior	5.8	1.3	3.2-8.4	5.0	1.1	2.8-7.2
	septal	5.8	1.3	3.2-8.4	5.0	1.1	2.8-7.2
	lateral	5.5	1.3	2.9-8.1	4.6	1.1	2.4-6.8
	**mean**	5.6	1.0	3.6-7.6	4.7	0.9	2.9-6.5

* = calculated as mean ± (2*SD).

**Table 28:** **Normal left ventricular myocardial thickness in mm measured on short axis images for men and women**

		**Men**			**Women**		
**Level**	**Segment**	**mean**_**p**_	**SD**_**p**_	Lower/upper limits*	**mean**_**p**_	**SD**_**p**_	Lower/upper limits*
basal	1	8.2	1.1	6.0-10.4	6.7	1.0	4.7-8.7
	2	9.6	1.1	7.4-11.8	7.9	1.0	5.9-9.9
	3	9.2	1.1	7.0-11.4	7.5	1.0	5.5-9.5
	4	8.1	1.1	5.9-10.3	6.6	1.0	4.6-8.6
	5	7.3	1.1	5.1-9.5	6.0	1.0	4.0-8.0
	6	7.4	1.1	5.2-9.6	6.1	0.9	4.3-7.9
mid-cavity	7	6.7	1.1	4.5-8.9	5.7	1.0	3.7-7.7
	8	7.7	1.1	5.5-9.9	6.4	1.0	4.4-8.4
	9	8.2	1.1	6.0-10.4	6.9	1.0	4.9-8.9
	10	7.0	1.1	4.8-9.2	5.9	1.0	3.9-7.9
	11	6.2	1.1	4.0-8.4	5.2	0.9	3.4-7.0
	12	6.4	1.1	4.2-8.6	5.4	1.0	3.4-7.4
apical	13	6.7	1.1	4.5-8.9	6.4	1.0	4.4-8.4
	14	7.3	1.1	5.1-9.5	6.3	1.0	4.3-8.3
	15	6.2	1.1	4.0-8.4	5.4	1.0	3.4-7.4
	16	6.3	1.1	4.1-8.5	5.9	1.0	3.9-7.9

Pooled weighted mean values from references [29,30].

mean_p_ = pooled weighted mean; SD_p_ = pooled standard deviation; * = calculated as mean_p_ ± 2*SD_p_; Segments: 1 = basal anterior, 2 = basal anteroseptal, 3 = basal inferoseptal, 4 = basal inferior, 5 = basal inferolateral, 6 = basal anterolateral, 7 = mid anterior, 8 = mid anteroseptal, 9 = mid inferoseptal, 10 = mid inferior, 11 = mid inferolateral, 12 = mid anterolateral, 13 = apical anterior, 14 = apical septal, 15 = apical inferior, 16 = apical lateral.

## Cardiac valves and quantification of flow

### CMR acquisition parameters

Prospectively and retrospectively ECG- gated phase contrast (PC) CMR sequences are available on most CMR machines. Prospectively-gated sequences use arrhythmia rejection and may be performed in a breath hold. Retrospectively gated techniques are mainly performed during free-breathing, often with higher spatial and temporal resolution compared to the breath hold techniques [31]. 4D PC flow quantification techniques show initial promising results, but 2D PC flow techniques are currently used in the daily clinical routine [32]. Apart from PC-CMR, valve planimetry—using ECG-gated SSFP CMR— can also be used to estimate stenosis or insufficiencies with good correlation to echocardiographic measurements [33].

Measurements of flow are most precise when a) the imaging plane is positioned perpendicular to the vessel of interest and b) the velocity encoded gradient echo (V_enc_) is encoded in a through plane direction [34]. The slice thickness should be <7 mm to minimize partial volume effects. Compared to aortic or pulmonary flow evaluation, quantification of mitral or tricuspid valves is more challenging using PC-CMR due to substantial through plane motion during the cardiac cycle [35].

#### Flow encoding velocity (V_enc_)

The V_enc_ should be chosen close to the maximum expected flow velocity of the examined vessel for precise measurements. Setting the V_enc_ below the peak velocity results in aliasing. For the normal aorta and main pulmonary artery, maximum velocities do not exceed 150 and 90 cm/sec, respectively.

Adequate temporal resolution is necessary to avoid temporal flow averaging, especially for the evaluation of short, fast, and turbulent jets within a vessel (e.g. aortic stenosis). For the clinical routine, 25–30 msec temporal resolution is usually sufficient. The minimum required spatial resolution should be less than one third of the vessel diameter to avoid partial volume effects with the adjacent vessel wall and surrounding stationary tissues for small arteries [34].

### CMR analysis methods

For data analysis, dedicated flow software should be used. Most of the currently available flow software tools offer semi-automatic vessel contouring, which needs to be carefully checked by the examiner.

The modified Bernoulli equation (∆P = 4 × V_max_^2^) is commonly used for calculation of pressure gradients using PC-CMR across the pulmonary or aortic valve [36,37].

It has to be considered that velocity measurements of a stenotic lesion with high jet velocities might be inaccurate due to partial volume effects in case of a small jet width and also the limited temporal resolution compared to the high velocity of the jet. Measurements are further affected by signal loss due to the high velocity that may lead to phase shift errors and dephasing. Misalignement of the slice relative to the direction of the jet may lead to an underestimation of the peak velocity [38].

### Demographic parameters

To our knowledge, data of the association between normal values of flow and valve planimetry with demographic parameters has not been previously published.

### Studies included in this review

There is good agreement between PC-CMR, SSFP CMR planimetry, and echocardiography measurements, American Heart Association (AHA) criteria for grading valve stenosis or insufficiency is suggested [33,39,40] (Table 29). To date, there is no publication of normal reference values of flow and valve planimetry based on CMR measurements.

**Table 29:** **Grading valve disease adapted from echocardiography** [39,41]

**Valve disease**	**Indicator**	**Mild**	**Moderate**	**Severe**
Aortic stenosis	Peak velocity [m/s]	<3	3-4	>4
	Orifice area [cm^2^]	>1.5	1.0-1.5	<1.0
	Orifice area /BSA [cm^2^/m^2^]			<0.6
Aortic regurgitation	Regurgitant volume [ml/beat]	<30	30-59	≥60
	Regurgitant fraction [%]	<30	30-49	≥50
	Regurgitant orifice area [cm^2^]	<0.10	0.10-0.29	≥0.30
Mitral stenosis	Peak velocity [m/s]	<1.2	1.2-2.2	>2.2
	Orifice area [cm^2^]	>1.5	1.0-1.5	<1.0
Mitral regurgitation	Regurgitant volume [ml/beat]	<30	30-59	≥60
	Regurgitant fraction [%]	<30	30-49	≥50
	Regurgitant orifice area [cm^2^]	<0.20	0.20-0.39	≥0.40
Pulmonary stenosis	Peak velocity [m/s]	<3	3-4	>4
	Orifice area [cm^2^]			<1
Pulmonary regurgitation	Regurgitant volume [ml/beat]	<30	30-40	>40
	Regurgitant fraction [%]	<25	20-35	>35
Tricuspid stenosis	Orifice area [cm^2^]			<1.0

Mitral valve flow velocities and deceleration time as for determination of diastolic left ventricular function measured by CMR showed a good correlation with measurements derived by transthoracic echocardiography but with a systematic underestimation [42] (Table 30).

**Table 30:** **Mitral valve flow for determination of diastolic left ventricular function according to reference** [43]

**Parameter**	**Normal**	**Type 1 (Impaired relaxation)**	**Type 2 (Pseudonormal)**	**Type 3 (Restrictive, partially reversible)**	**Type 3 (Restrictive, fixed)**
MDT (ms)	150-220	Increased	Normal	Decreased	Decreased
E/A ratio	1-2	<1	1-2	>2	>2

MDT = mitral deceleration time; E/A ratio = ratio of the mitral early (E) and atrial (A) components of the mitral inflow velocity profile.

## Normal aortic dimensions in the adult

### CMR acquisition parameters

Three- dimensional contrast enhanced MR Angiography (MRA) has gained broad acceptance and is widely used for assessment and follow-up of thoracic aortic diameter in clinical setting. The multi-planar reformation of MRA images leads to an accurate measurement perpendicular to the lumen of the vessel. However, the need of a contrast injection is a limitation for the use of this technique in patients who need multiple follow up examinations and in population based study settings [44]. Alternatively non-contrast techniques such as ECG gated non contrast 3D (2D) balanced steady state free precession (SSFP) CMR can be applied. The modulus image of phase contrast CMR has also been used to measure diameters of the aorta [45]. 2D Black blood CMR is used for a more detailed aortic wall assessment. In 2D acquisitions, the imaging plane needs to be acquired correctly at the time of the scan; thus any alterations in the imaging plane will result in a higher variability and lower accuracy of measurements. Another limitation for ascending thoracic aorta diameter measurement is the through plane motion during the cardiac cycle which can be minimized with ECG gating [44]. Potthast and colleagues compared the diameter of the ascending aorta obtained by different CMR sequences to ECG-triggered CT angiography as the gold standard and reported that ECG gated navigator triggered 3D SSFP sequence showed the best agreement with CT [44].

### CMR analysis methods

**Figure 13:**
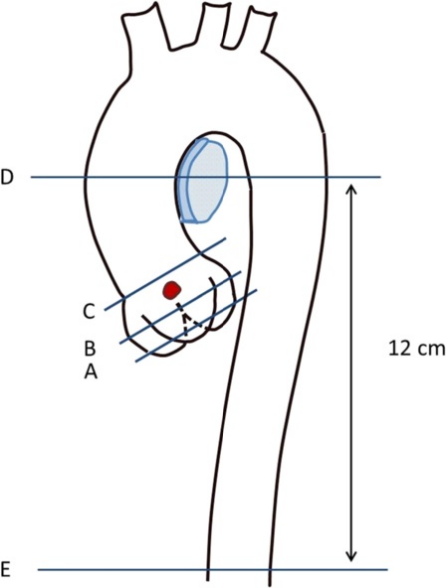
**The anatomic locations of aorta measurements: A. aortic valve annulus; B. aortic sinus; C. sinotubular junction; D. ascending aorta and proximal descending aorta; E. abdominal aorta.**

It is important to indentify the anatomic locations of diameter measurements of the thoracic aorta. In the studies cited here, measurements were obtained at the following anatomic locations: 1. aortic root cusp-commissure and cusp-cusp measurements; 2. aortic valve annulus; 3. aortic sinus; 4. sinotubular junction; 5. ascending aorta and proximal descending aorta: measurements at the level of the right pulmonary artery; 6. abdominal aorta: 12 cm distal to the pulmonary artery (Figure 13).

**Figure 14:**
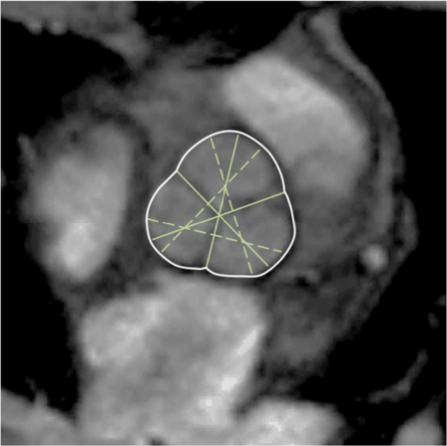
**Cusp-commissure (continuous lines) and cusp-cusp (dashed-lines) measurements at the level of the aortic sinus.**

The sagittal oblique view of the left ventricular outflow tract was used for measuring diameter at the level of the aortic annulus, the aortic sinus, and the sinotubular junction. Axial cross sectional images at predefined anatomic levels were used for measuring the ascending and descending aorta [46] as well as cusp-commissure and cusp-cusp diameters at the level of the aortic sinus [47] (Figures 13 and 14). There is no definite convention about measuring the luminal or outer to outer diameter of the aorta. Usually, measurement technique depends on the resolution and characteristics of the available MRI sequence. In the tables below, the method is specified.

### Demographic parameters

Age, gender and body size are major determinants of physiologic variation in aortic size. In the Multi-Ethnic Study of Atherosclerosis, which included participants from four different ethnicities, the race/ethnicity were not clinically significant determinants of ascending aorta diameter [45].

Aortic diameter and ascending aorta length increase with age, leading to decreased curvature of the aortic arch [48,49]. The association of age with aortic diameter was more marked in the ascending aorta compared to the descending thoracic and abdominal aorta, respectively [50,51]. Additionally, the descending aorta did not demonstrate age associated lengthening [49].

### Studies included in this review

Studies with normal values of aortic diameters including 50 or more subjects for both men and women and a range of ages (due to the age dependence of aortic diameters) have been included in this review (Tables 31, 32, 33, 34 and 35). There are three major publications regarding MR-based measurements of the thoracic aorta in adults: Davis et al. determined aortic diameter at three levels (ascending aorta, proximal descending aorta and abdominal aorta) by calculating the luminal diameter based on measurements of the cross sectional area obtained on cine SSFP images [46]. In the original publication normal age and gender specific absolute and indexed (for BSA) values are presented in a graph and absolute numbers are presented for different weight categories (Table 32).

**Table 31:** **References, normal aortic dimensions in adults**

**First author, year**	**CMR technique**	**N, male: female**	**Age range (yrs)**
Davis, 2014 [46]	SSFP; luminal diameter of thoracic and abdominal aorta	208: 239	19-70
Turkbey, 2013 [45]	Phase contrast (magnitude image); luminal diameter of ascending thoracic aorta	770:842	45-84
Burman, 2008 [47]	SSFP; luminal diameter of aortic root	60:60	20-80

SSFP = steady-state free precession; yrs = years.

**Table 32:** **Normal values (in mm) of the thoracic and abdominal aortic luminal diameters for men and women of different BMI categories measured at diastole (mean[±2SD]) according to** [46]

	**Men**			**Women**		
**Level**	**Normal weight**	**Overweight**	**Obese**	**Normal weight**	**Overweight**	**Obese**
**Aortic annulus**	23.9 (18.6-29.2)	24.3 (18.9-29.7)	25.6 (20.4-30.8)	20.6 (17.4-23.8)	21.7 (18.4-25.0)	21.5 (17.2-25.8)
**Aortic sinus**	31.9 (24.3-39.5)	32.8 (25.2-40.4)	33.3 (24.3-42.3)	27.5 (21.9-33.1)	28.0 (21.8-34.2)	27.5 (21.3-33.7)
**Sinotubular junction**	24.4 (18.2- 30.6)	25.7 (16.7- 34.7)	26.2 (18.9- 33.5)	21.6 (16.6- 26.6)	22.3 (17.0- 27.6)	22.1 (15.9- 28.3)
**Ascending aorta**	26.0 (18.7-33.3)	27.4 (18.9-35.9)	28.5 (23.1-33.9)	24.7 (17.8-31.6)	26.5 (19.3-33.7)	26.6 (18.8-34.4)
**Prox. desc. aorta**	20.1 (14.7-25.5)	20.9 (15.6-26.2)	22.2 (16.3-28.1)	18.5 (14.6-22.4)	19.2 (14.8-23.6)	19.6 (16.5-23.2)
Abdominal aorta *	17.1 (12.0-22.2)	17.9 (12.8-23.0)	18.8 (14.4-23.2)	16.0 (12.1-19.9)	16.3 (12.3-20.3)	17.4 (13.9-20.9)

normal weight = BMI <25 kg/m^2^; overweight = BMI 25–29.9 kg/m^2^; obese = BMI >30 kg/m^2^; prox. desc. aorta = proximal descending aorta; * = abdominal aorta measured 12 cm distal to the pulmonary artery.

**Table 33:** **Absolute and BSA indexed normal values of ascending aortic luminal diameter for men and women of different age categories (median [5th-95th percentile]) measured on phase contrast images according to** [45]

**Age (years)**	**Men**	**Women**
**Absolute values (mm)**		
**45-54**	31.6 (27.2-37.3)	28.8 (24.6-34.4)
**55-64**	32.8 (28.1-40.7)	30.1 (25.7-36.4)
**65-74**	34.2 (28.7-41.0)	30.6 (26.1-36.3)
**75-84**	34.7 (28.6-40.8)	31.1 (26.8-37.1)
**Values indexed to BSA (mm/m** ^**2**^ **)**		
**45-54**	15.9 (13.3-19.5)	16.7 (13.5-20.7)
**55-64**	16.8 (13.6-21.1)	17.6 (14.8-22.1)
**65-74**	17.8 (14.2-21.8)	18.1 (14.5-22.1)
**75-84**	18.6 (15.2-22.6)	19.7 (15.3-28.2)

BSA = body surface area.

**Table 34:** **Absolute and indexed (to BSA) normal values of aortic root cusp-commissure measurements for men and women of different age categories measured at systole and diastole (mean ± SD [lower/upper limits calculated as mean ± 2SD]) according to** [47]

	**Men**		**Women**	
**Age (years)**	**systolic**	**diastolic**	**systolic**	**diastolic**
**Absolute values (mm)**				
**20-29**	32.6 ± 3.5 (26–40)	30.4 ± 3.3 (24–37)	28.6 ± 3.9 (21–36)	26.3 ± 3.9 (19–34)
**30-39**	32.0 ± 3.3 (25–39)	29.7 ± 3.5 (23–37)	28.5 ± 2.8 (23–34)	26.8 ± 2.8 (21–32)
**40-49**	33.3 ± 2.1 (29–38)	31.6 ± 1.6 (28–35)	31.7 ± 2.8 (26–37)	30.0 ± 2.1 (26–34)
**50-59**	33.9 ± 5.1 (24–44)	32.7 ± 4.8 (23–42)	29.5 ± 2.0 (26–34)	28.4 ± 1.8 (25–32)
**60-69**	34.6 ± 2.5 (30–40)	33.5 ± 2.3 (29–38)	30.5 ± 1.9 (27–34)	29.5 ± 2.0 (26–34)
**70-79**	35.1 ± 3.1 (29–41)	33.9 ± 3.0 (28–40)	30.7 ± 1.3 (28–33)	29.6 ± 1.4 (27–32)
**all**	33.6 ± 3.4 (27–40)	32.0 ± 3.5 (25–39)	29.9 ± 2.7 (25–35)	28.4 ± 2.8 (23–34)
**Values indexed to BSA (mm/m** ^**2**^ **)**				
**20-29**	16.8 ± 1.6 (14–20)	15.6 ± 1.7 (12–19)	16.7 ± 1.9 (13–21)	15.3 ± 2.0 (11–19)
**30-39**	16.3 ± 1.6 (13–20)	15.1 ± 1.6 (12–18)	17.5 ± 1.3 (15–20)	16.4 ± 1.3 (14–19)
**40-49**	16.1 ± 1.1 (14–18)	15.3 ± 1.0 (13–17)	17.8 ± 2.6 (13–23)	16.8 ± 2.3 (12–21)
**50-59**	17.2 ± 2.1 (13–21)	16.6 ± 1.9 (13–20)	17.8 ± 1.4 (15–21)	17.2 ± 1.4 (14–20)
**60-69**	17.7 ± 1.8 (14–21)	17.2 ± 1.7 (14–21)	17.7 ± 1.5 (15–21)	17.1 ± 1.4 (14–20)
**70-79**	18.0 ± 1.2 (16–20)	17.4 ± 1.2 (15–20)	18.5 ± 0.9 (17–20)	17.8 ± 0.9 (16–20)
**all**	17.0 ± 1.7 (14–20)	16.2 ± 1.8 (13–20)	17.7 ± 1.7 (14–21)	16.8 ± 1.7 (13–20)

BSA = body surface area.

**Table 35:** Absolute and indexed (to BSA) normal values of aortic root cusp-cusp measurements for men and women of different age categories measured at systole and diastole (mean ± SD [lower-upper limits calculated as mean ± 2*SD]) according to [47]

	**Men**		**Women**	
**Age (years)**	**systolic**	**diastolic**	**systolic**	**diastolic**
**Absolute values (mm)**				
**20-29**	34.4 ± 4.2 (26–43)	32.8 ± 3.8 (25–40)	30.2 ± 4.7 (21–40)	28.4 ± 4.7 (19–38)
**30-39**	33.8 ± 3.8 (26–41)	32.0 ± 3.9 (24–40)	30.0 ± 3.1 (24–36)	28.7 ± 3.0 (23–35)
**40-49**	36.0 ± 2.7 (31–41)	34.1 ± 2.3 (30–39)	33.9 ± 2.5 (29–39)	32.8 ± 2.5 (28–38)
**50-59**	36.3 ± 5.9 (25–48)	35.2 ± 5.7 (24–47)	31.4 ± 2.5 (26–36)	30.6 ± 2.6 (25–36)
**60-69**	37.4 ± 2.9 (32–43)	36.2 ± 2.5 (31–41)	32.8 ± 2.3 (28–37)	32.0 ± 2.2 (28–36)
**70-79**	37.8 ± 3.9 (30–46)	37.0 ± 3.5 (30–44)	32.9 ± 1.7 (30–36)	32.0 ± 1.6 (29–35)
**all**	36.0 ± 4.1 (28–44)	34.6 ± 4.0 (27–43)	31.9 ± 3.2 (26–38)	30.7 ± 3.3 (24–37)
**Values indexed to BSA (mm/m** ^**2**^ **)**				
**20-29**	17.7 ± 1.9 (14–22)	16.9 ± 1.9 (13–21)	17.6 ± 2.3 (13–22)	16.6 ± 2.3 (12–21)
**30-39**	17.2 ± 2.0 (13–21)	16.2 ± 1.9 (12–20)	18.4 ± 1.4 (16–21)	17.6 ± 1.3 (15–20)
**40-49**	17.4 ± 1.4 (15–20)	16.5 ± 1.3 (14–19)	19.0 ± 2.7 (14–24)	18.4 ± 2.5 (13–23)
**50-59**	18.5 ± 2.4 (14–23)	17.9 ± 2.3 (13–23)	18.9 ± 1.7 (16–22)	18.5 ± 1.8 (15–22)
**60-69**	19.2 ± 2.2 (15–24)	18.6 ± 2.0 (15–23)	19.0 ± 1.8 (15–23)	18.6 ± 1.6 (15–22)
**70-79**	19.4 ± 1.4 (17–22)	19.0 ± 1.3 (16–22)	19.8 ± 1.0 (18–22)	19.3 ± 1.0 (17–21)
**all**	17.6 ± 2.0 (14–22)	17.5 ± 2.0 (14–22)	18.8 ± 1.9 (15–23)	18.1 ± 2.0 (14–22)

BSA = body surface area.

Turkbey et al. measured the luminal diameter of the ascending aorta on magnitude images of a phase contrast sequence in a large number of healthy subjects [45] (Table 33).

Burmann et al. performed detailed measurements of the aortic root including cusp-commissure and cusp-cusp measurements at diastole and systole on cine SSFP images [47] (Tables 34 and 35).

## Normal aortic dimensions in children

### CMR acquisition parameters

There is no consensus regarding the sequence type used to measure aortic diameters and areas. In the three major publications (Table 36) measurements were obtained on 3 dimensional contrast enhanced MR angiography [52], gradient echo images [53] and phase contrast cine images [54].

**Table 36:** **References, normal aortic dimensions in children**

**First author, year**	**CMR technique**	**N, male: female**	**Age range (yrs)**
Kaiser, 2008 [52]	Contrast enhanced 3D MR angiography; shortest diameter	30:23	2-20
Voges, 2012 [53]	Cine GRE; measurements obtained at maximal distension of the aorta	30:41	2.3-28.3
Kutty, 2012 [54]	Cine phase contrast CMR; measurements obtained at systole	55:50	4.4-20.4

GRE = gradient echo; 3D = 3-dimensional; yrs = years.

### CMR analysis methods

In order to reduce error in measurement, care has to be taken to obtain or reconstruct cross sectional images that are true perpendicular instead of oblique to the course of the vessel. Kaiser et al. demonstrated that aortic diameter measurements show a slight variation with measurement plane with a mean difference between measurements on cross-sectional and longitudinal images of 0.16 mm and a coefficient of variability of 2.1% [52].

Kutty et al. indicate that in their study the outer diameter of the vessel was measured [54] while Kaiser et al. and Voges et al. do not mention details in this respect [52,53].

### Demographic parameters

Aortic diameters vary by BSA [52,54] but do not show gender differences [53,54]. Aortic area did also not show any gender differences [53].

### Studies included in this review

There are three publications of a systematic evaluation of aortic dimensions (diameter and/or area) in children that vary by CMR-technique, measurement technique and data presentation (Table 36): In the study by Kaiser et al. aortic diameter was measured as the shortest diameter passing the center of the vessel at 9 levels of the thoracic aorta on reconstructed cross-sectional images of a contrast enhanced 3 dimensional MR angiography [52]. In the original publication data is presented as median and range as well as percentiles, z-scores and regression models incorporating BSA. Voges et al. present measurements obtained at four levels of the thoracic aorta obtained on cine GRE images at maximal aortic distension as mean ± standard deviation and as percentiles [53]. In the study by Kutty et al. aortic diameter and area was measured 1-2 cm distal to the sinotubular junction at systole on phase contrast cine images [54]. Data is presented as mean ± standard deviation, regression equation and z-scores.

**Figure 15:**
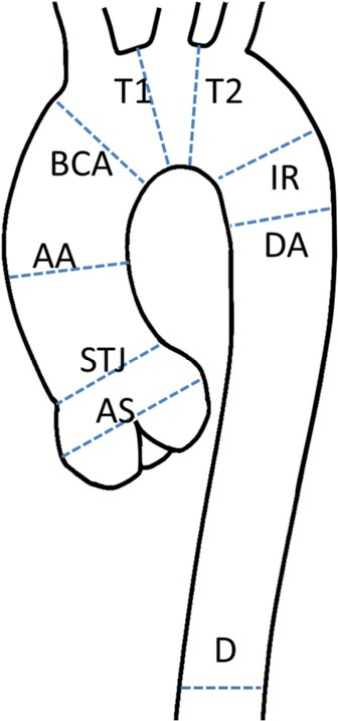
**Sites of measurement.** AS = aortic sinus; STJ = sinotubular junction; AA = ascending aorta; BCA = proximal to the origin of the brachiocephalic artery; T1 = first transverse segment; T2 = second transverse segment; IR = isthmic region; DA = descending aorta; D = thoracoabdominal aorta at the level of the diaphragm.

**Figure 16:**
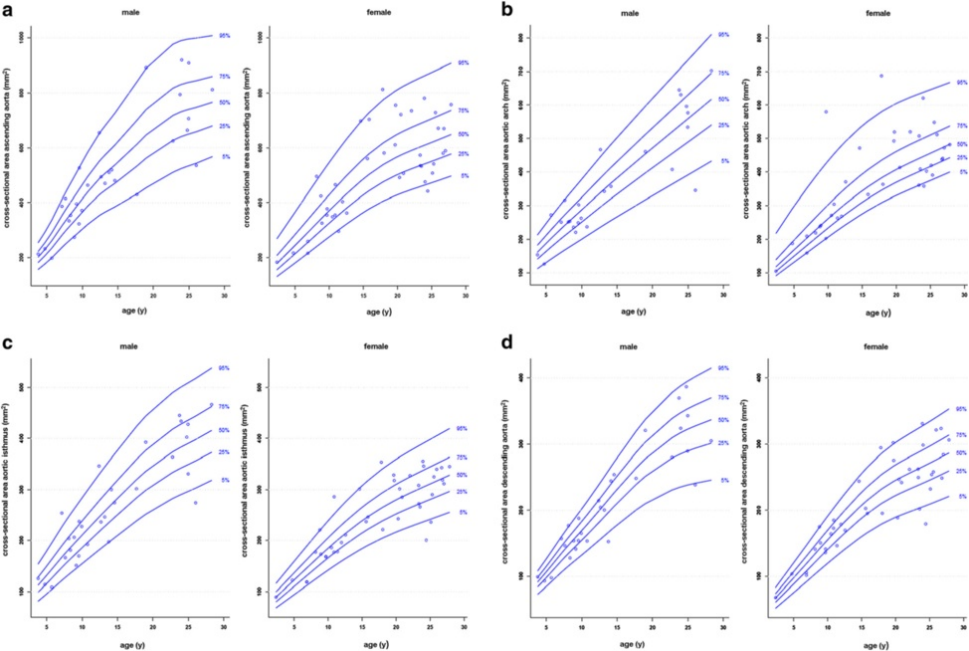
**Reference percentiles for aortic areas measured at four different sites (ascending aorta [a], aortic arch [b], aortic isthmus [c] and descending aorta above the diaphragm [d]) on cine GRE images at maximal aortic distension according to reference [** 53 **].**

In this review we present regression equations of normal aortic diameters measured at 9 different sites according to [52] (Table 37, Figure 15) and of normal area of the ascending aorta according to [54] (Table 38). Further reference percentiles of aortic area measured at 4 different locations obtained on cine GRE images are presented in Figure 16 according to [53]. The z-scores for each aortic diameter (D) can be calculated with the following equation:

**Table 37:** **Normal aortic diameters (in mm) measured on reconstructed cross-sectional images of a contrast enhanced 3D-MR angiography according to reference** [52]

**Site**	**Predicted diameter**	**SD of residuals**
Aortic sinus	0.57 + 19.37*BSA^0.5^	2.38
Sinotubular junction	−0.03 + 16.91*BSA^0.5^	1.92
Ascending aorta	−1.33 + 18.6*BSA^0.5^	1.99
Proximal to the origin of the brachiocephalic artery	−3.38 + 20.07*BSA^0.5^	1.69
First transverse segment	−3.52 + 18.66*BSA^0.5^	1.63
Second transverse segment	−2.63 + 16.5*BSA^0.5^	1.31
Isthmic region	−3.37 + 16.52*BSA^0.5^	1.46
Descending aorta	−1.12 + 14.42*BSA^0.5^	1.64
Thoracoabdominal aorta at the level of the diaphragm	1.27 + 9.89*BSA^0.5^	1.34

BSA = body surface area; SD = standard deviation.

**Table 38:** **Normal aortic area (in cm** ^**2**^ **) measured 1-2 cm distal to the sinotubular junction at systole on phase contrast cine images according to reference** [54]

**Site**	**Predicted diameter**
Ascending aorta	−0.0386 + 2.913*BSA

BSA = body surface area.

$$ \mathrm{z}\hbox{-} \mathrm{score} = \left(\mathrm{measured}\ \mathrm{D}\ \hbox{--}\ \mathrm{predicted}\ \mathrm{D}\right)/\mathrm{S}\mathrm{D}\ \mathrm{of}\ \mathrm{residuals} $$

on the base of the data provided in Table 37.

Due to the differences in acquisition and measurement technique as well as presentation of results, weighted mean values were not calculated.

## Normal aortic distensibility/ pulse wave velocity in adults

### CMR acquisition parameters

Pulse wave velocity (PWV) calculations using a velocity-encoded CMR with phase contrast sequences allow accurate assessment of aortic systolic flow wave and the blood flow velocity. The sequence should be acquired at the level of the bifurcation of the pulmonary trunk, perpendicular to both, the ascending and descending aorta. The distance between two aortic locations (aortic length) can be estimated from axial and coronal cine breath hold SSFP sequences covering the whole aortic arch [55]. Alternatively, sagittal oblique views of the aortic arch can be acquired using a black blood spin echo sequence [51].

Another measurement method of aortic stiffness is aortic distensibility. The cross sectional aortic area at different phases of the cardiac cycle is measured using ECG-gated SSFP cine imaging to assess aortic distensibility by CMR. Modulus images of cine phase contrast CMR can be used as well [56].

### CMR analysis methods

**Figure 17:**
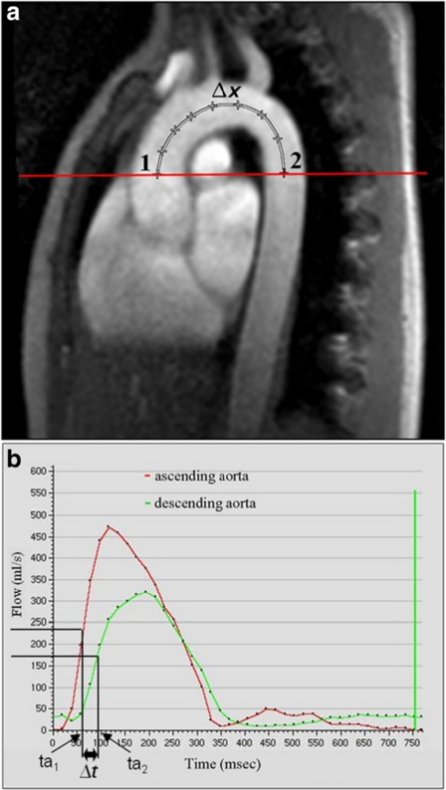
**Measurement of pulse wave velocity according to reference** **[** 53 **]** **.** Δx = length of the centerline between the sites of flow measurement in the ascending and descending aorta **(A)**; Δt = time delay between the flow curves obtained in the descending aorta relative to the flow curve obtained in the ascending aorta calculated between the midpoint of the systolic up slope tails on the flow versus time curves of the ascending aorta (ta1) and the descending aorta (ta2) **(B)**.

PWV is the most validated method to quantify arterial stiffness using CMR. PWV is calculated by measuring the pulse transit time of the flow curves (Δt) and the distance (D) between the ascending and descending aortic locations of the phase contrast acquisition [51]: Aortic PWV = D/ Δt (Figure 17).

PWV increases with stiffening of arteries since the stiffened artery conducts the pulse wave faster compared to more distensible arteries.

Aortic distensibility is calculated with the fallowing formula after measuring the minimum and maximum aortic cross sectional area [57]:

Aortic Distensibility = (minimum area- maximum area)/ (minimum area x ΔP x 1000) where ΔP is the pulse pressure in mmHg.

### Demographic parameters

Greater ascending aorta diameter and changes in aortic arch geometry by aging was significantly associated with increased regional stiffness of the aorta, especially the ascending portion. The relationship of age with measures of aortic stiffness is non –linear and the decrease of aortic distensibility is steeper before the fifth decade of life [51]. Males have stiffer aortas compared to females [58].

### Studies included in this review

Two publications reported normal values of pulse wave velocity and aortic distensibility (Tables 39, 40 and 41).

**Table 39:** **References, ascending and descending thoracic aorta distensibility, aortic arch pulse wave velocity**

**First author, year**	**CMR technique**	**N, male: female**	**Age range (yrs)**
Redheuil, 2010 [51]	Phase contrast CMR and gradient echo cine	54:57	20-84
Rose, 2010 [58]	SSFP	13:13	23-61

yrs = years; SSFP = steady-state free precession.

**Table 40:** **Normal values of ascending and descending thoracic aorta distensibility and aortic arch pulse wave velocity by age categories (mean ± SD) according to** [51]

	**Age categories (years)**					
	**20-29**	**30-39**	**40-49**	**50-59**	**60-69**	**≥70**
**Ascending aortic distensibility (kPa** ^**−1**^ **. 10** ^**−3**^ **)**	74 ± 23	61 ± 23	31 ± 18	18 ± 7	12 ± 7	10 ± 6
**Descending aortic distensibility (kPa** ^**−1**^ **. 10** ^**−3**^ **)**	72 ± 18	70 ± 24	38 ± 17	29 ± 13	18 ± 8	17 ± 6
**Aortic arch PWV (m/s)**	3.5 ± 0.5	3.9 ± 1.1	5.6 ± 1.4	7.2 ± 2.3	9.7 ± 2.9	11.1 ± 4.6

PWV = Pulse wave velocity.

**Table 41:** **Normal values of ascending and descending thoracic aorta distensibility (in 10** ^**−3**^ **mmHg** ^**−1**^ **) by gender (mean ± SD) according to** [58]

	**Men**	**Women**
**Ascending Aorta**	6.1 ± 2.5	8.6 ± 2.7
**Descending Aorta**	5.1 ± 2.4	7.2 ± 1.6

## Normal aortic distensibility/ pulse wave velocity in children

### CMR acquisition parameters

In the only publication of aortic distensibility and pulse wave velocity in children, distensibility was measured on gradient echo cine CMR images and pulse wave velocity was measured on phase-contrast cine CMR [53].

### CMR analysis methods

Distensibility was calculated as (A_max_ – A_min_)/A_min_ x (P_max_ – P_min_), where A_max_ and A_min_ represent the maximal and minimal cross-sectional areal of the aorta, and P_max_ and P_min_ represent the systolic and diastolic blood pressure measured with a sphygmomanometer cuff around the right arm.

Pulse wave velocity was calculated as Δx/Δt, where Δx is defined as the length of the centerline between the sites of flow measurement in the ascending and descending aorta and Δt represents the time delay between the flow curve obtained in the descending aorta relative to the flow curve obtained in the ascending aorta (Figure 17).

### Demographic parameters

Aortic distensibility and pulse wave velocity did not vary by gender. Aortic distensibility decreases with age and correlates with height, body weight and BSA [53].

### Studies included in this review

**Figure 18:**
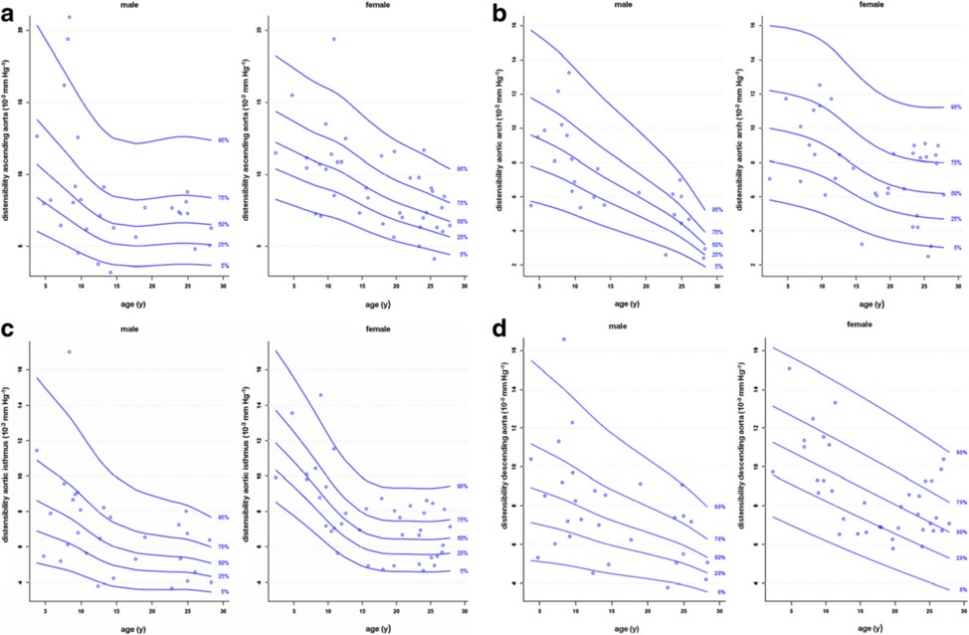
**Reference percentiles for aortic distensibility measured at four different sites (ascending aorta [a], aortic arch [b], aortic isthmus [c] and descending aorta above the diaphragm [d]) on cine GRE images at maximal aortic distension according to reference [** 53 **].**

**Figure 19:**
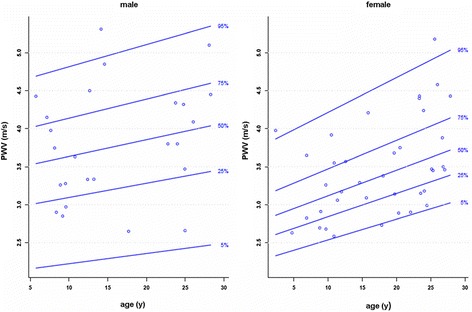
**Reference percentiles for aortic pulse wave velocity according to reference [** 53 **].**

There is a single publication only of a systematic evaluation of normal aortic distensibility and pulse wave velocity in children (Table 42). Reference percentiles by age according to reference [53] are presented in Figures 18 and 19.

**Table 42:** **References, normal aortic distensibility and pulse wave velocity in children**

**First author, year**	**CMR technique**	**N, male: female**	**Age range (yrs)**
Voges, 2012 [53]	Cine GRE; phase-contrast cine CMR	30:41	2.3-28.3

GRE = gradient echo; yrs = years.

## Normal values of myocardial T1 relaxation time and the extracellular volume (ECV)

### CMR acquisition parameters

Most of the published myocardial T1 values have been acquired using a Modified Look-Locker Inversion Recovery (MOLLI) [59] or shortened-MOLLI (ShMOLLI) [60] method, combined with a balanced SSFP read out [59]. The MOLLI method acquires data over 17 heartbeats with a 3(3bt)3(3bt)5 sampling pattern, while ShMOLLI has been described with a 9 heart beat breath-hold and a 5(1bt)1(1bt)1 sampling pattern, although variations of these acquisition schemes have been proposed [60]. An alternative sampling method is saturation recovery single-shot acquisition (SASHA) in which a first single-shot bSSFP image is acquired without magnetization preparation followed by nine images prepared with variable saturation recovery times [61]. All methods usually acquire images at end diastole to limit cardiac motion artifacts [59] but acquisition of T1 maps at systole has been shown to be feasible [62]. Post contrast T1 values have been performed following a bolus or primed infusion (Equilibrium-EQCMR) with good agreement of ECV values up to 40% [63].

#### Factors affecting T1 and ECV

Field strength has a significant effect on T1 values; with 3T scans producing 28% higher native T1 and 14% higher post contrast T1 values when compared with 1.5T [62]. Post contrast T1 is also affected by the dose and relaxivity of the contrast agent used, contrast clearance, and the time between injection and measurement [62,64,65]. There is also greater heterogeneity for a T1 native normal range at 3 Tesla [62,66,67]. Further, it has been shown that T1 varies by cardiac phase (diastole versus systole) and region of measurement (septal versus non-septal) [62]. ECV values are relatively unaffected by field strength (3T versus 1.5T). Both native T1 and ECV values have been shown to be less reliable in the infero-lateral wall [62,68].

Flip-angle and pre-pulse can also affect normal values, with the adiabatic pre-pulse increasing native T1 values by approximately 25 ms compared with non-adiabatic pre-pulses. FLASH mapping sequences produce significantly lower native T1 values than bSSFP methods [69,70].

### CMR analysis methods

T1 maps are based on pixel-wise quantification of longitudinal relaxation of the acquired images. Native T1 measures a composite signal from myocytes and interstitium and is expressed in ms [71]. Measurements that correlate pre and post contrast T1 myocardial values and blood T1 have been proposed, such as the partition coefficient or the extracellular volume fraction (ECV), expressed as a percantage [72].

**Figure 20:**
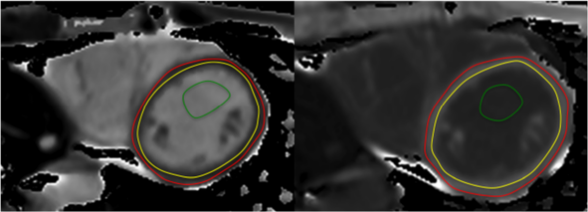
**T1 maps pre- and post-contrast with left ventricular endocardial and epicardial contours.**

Offline post-processing involves manually tracing endocardial and epicardial contours [65,73] (Figure 20) or placing a region of interest within the septal myocardium using a prototype tool [67]. Inclusion of blood pool or adjacent tissue should be carefully avoided. Motion correction is generally used to counter undesired breathing motion. However, motion correction can only correct for in-plane motion and not through-plane motion. All methods, therefore, are vulnerable to partial volume effects. Some investigators also corrected for this with smaller regions of interest and co-registration of images [74].

### Demographic parameters

Increasing age has been shown to increase ECV in healthy volunteers in one publication [68] and females less than 45 years have been shown to have a higher precontrast T1 [74].

### Studies included in this review

Several studies have shown a strong correlation between T1 values by CMR and diffuse myocardial fibrosis on myocardial biopsy [71,75,76], but the rapid evolution of acquisition methods over the past years has led to inconsistent T1 values reported in the literature.

In order to reflect the current literature, the normal values presented here are classified by field strength and acquisition pulse sequence and list pre contrast, post contrast, and ECV values where available.

It should be noted that a universal normal range for T1 cannot be determined given the heterogeneity of acquisition pulse sequences used in the existing literature and because no true reference value for in vivo T1 exists. Table 43 is a summary of publications over the last years presenting normal values based on ≥ 20 healthy subjects as available in December 2013.

**Table 43:** **Normal native and post contrast T1 time and ECV (mean ± SD)**

**Author**	**n**	**Age (yrs)**	**Male (%)**	**Sequence**	**Gd type & dose (mmol/kg)**	**Time p.c. (min)**	**T1 native (ms)**	**T1 p.c. (ms)**	**ECV**	**Slice(s), % thickness**
**1.5 Tesla**										
Iles (2008) [77]	20	38 ± 3	50	GE, VAST 3–4 BH	0.2 Magnevist	15	975 ± 62	564 ± 23	n/a	3x SAX & 4Ch full thickness
Ugander (2012) [68]	60	49 ± 17	52	MOLLI	0.15-0.2 Magnevist	15-20	Not reported	Not reported	27 ± 3	1x SAX & 4Ch full thickness
Kellman (2012) [78]	62	44 ± 17	48	MOLLI	0.15 Magnevist	15-20	965 ± 35	Not reported	25 ± 3**	1x SAX & 4Ch midwall
Flett (2012) [69]	30	64 ± 13	67	FLASH, multi-BH	0.1 Dotarem	45-80 EQCMR	698 ± 86	408 ± 33	n/a	2x SAX septum
Fontana (2012) [76]	50	47 ± 7	53	ShMOLLI*	0.1 Dotarem	45-80 EQCMR	Not reported	Not reported	26 ± 3	2x SAX septum
Sado (2012) [70]	81	44 ± 17	52	FLASH, multi-BH	0.1 Dotarem	45-80 EQCMR	Not reported	Not reported	25 ± 4	2x SAX septum
Kawel (2012) [62]	23	28 ± 6	36	MOLLI	0.15 Magnevist	12	1003 ± 46	522 ± 34	28 ± 3	1x SAX full thickness
Piechnik (2013) [74]	342	38 ± 15	49	ShMOLLI*	No contrast given	n/a	962 ± 25	n/a	n/a	3x SAX full thickness
Bull (2013) [75]	33	62 ± 7	64	ShMOLLI	No contrast given	n/a	944 ± 16	n/a	n/a	1x SAX septal midwall
**3 Tesla**										
Kawel (2012) [65]	23	28 ± 6	31	MOLLI	0.15 Magnevist	12	1286 ± 59	538 ± 34	28 ± 3	1x SAX full thickness
Kawel (2012) [65]	23	28 ± 6	33	MOLLI	0.1 Multihance	12	As above	555 ± 33	27 ± 3	1x SAX full thickness
Puntmann (2013) [67]	21	38 ± 6	23	MOLLI*	0.2 Gadobutrol	15	1056 ± 27	454 ± 53	26 ± 5	1x SAX septal midwall
Puntmann (2013) [79]	30	43 ± 9	63	MOLLI*	0.2 Gadobutrol	10	1070 ± 55	402 ± 58	27 ± 5	1x SA septal midwall
Liu (2012) [80]	24	29 ± 6	33	MOLLI	0.15 Magnevist	12	1159 ± 39	Not reported	27 ± 3	1x SAX full thickness
Liu (2012) [80]	24	29 ± 6	33	MOLLI	0.1 Multihance	12	1159 ± 39	Not reported	26 ± 3	1x SAX full thickness

Age: mean ± standard deviation; yrs = years; n = number of subjects; Gd = Gadolinium; p.c. = post contrast; ECV = extracellular volume fraction; MOLLI = modified look-locker inversion recovery; ShMOLLI = shortened MOLLI; GE = gradient echo; VAST = variable sampling of k-space; BH = breath hold; LL = Look-locker; FLASH = fast low angle single shot recovery; SAX = short axis; 4Ch = 4 chamber; Hb = heart beat; EQCMR = equilibrium contrast cardiovascular magnetic resonance; * = adiabatic pre pulse; ** = automated motion correction and co-registration.

For SASHA, only limited normal values are available. T1 estimates based on SASHA are higher than with MOLLI methods. One study reported SASHA derived T1 values in 39 normal subjects of 1170 ± 9 ms at 1.5T [61].

We conclude that at present, normal native T1 values are specific to pulse sequences and scanner manufacturer. For diagnostic purposes it is most important to use a method with a tight normal range, good reproducibility and sensitivity to disease.

## Normal values of myocardial T2* relaxation time

### CMR acquisition parameters

Quantification of the T2* relaxation time plays an important role for estimation of myocardial iron overload [81]. For quantification of the myocardial T2* time, the gradient-echo T2* technique with multiple increasing TEs is preferred over the spin-echo T2 technique due to a greater sensitivity to iron deposition [82-84]. Usually a single-breath hold technique is used. Normal values and a grading system for myocardial iron overload are available for 1.5T [84].

### CMR analysis methods

**Figure 21:**
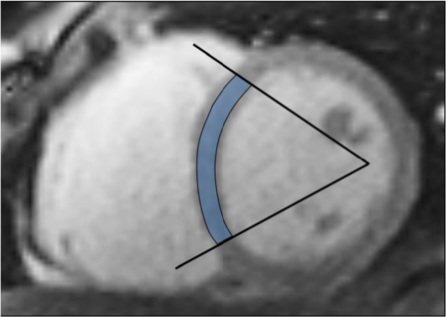
Measurements for myocardial T2* are obtained in the septum.

Since the gradient-echo T2* technique is vulnerable to distortions of the local magnetic field e.g. by air-tissue interfaces, measurements are obtained by placing a region of interest on the interventricular septum of a midventricular short axis slice, since the septum is surrounded by blood on both sides [83] (Figure 21).

T2* times are frequently reported as relaxation rate, representing the reciprocal of the time constant and calculated as R2* = 1000/T2*. The unit of R2* is Hertz or s^−1^ [83].

### Demographic parameters

It has been shown that T2* does not correlate with age [85]. To our knowledge the relationship between other demographic parameters and T2* has not been assessed yet.

### Studies included in this review

Generally a T2* value - measured at the interventricular septum using a multiecho GRE sequence at 1.5T - of >20 ms is considered normal while the mean myocardial T2* is around 40 ms [81].

Examples of studies that used the current multiecho GRE technique with a sample size of >10 healthy subjects are presented in Table 44.

**Table 44:** References (examples), normal myocardial T2* values

**First author, year**	**CMR technique**	**N, male: female**	**Age range (yrs)**	**Mean ± SD (ms)**
Kirk, 2010 [85]	GRE, 8 echo times (2.6-16.74 ms)	38:25	18-77	36.3*
Pepe 2006 [86]	GRE, 9 echo times (2.2-20.3)	14:6	(33 ± 9**)	36.4 ± 6.7
Anderson, 2001 [82]	GRE, 8 echo times (2.2-20.1 ms)	9:6	26-39	52 ± 16

GRE = gradient-echo; yrs = years; * = SD not indicated in original publication; ** = mean ± SD, range not mentioned in original publication.

Depending on the risk to develop heart failure as a consequence of myocardial iron overload, a grading system for disease severity has been published (Table 45) [87].

**Table 45:** Grading of iron overload based on T2* measurements according to [81,87]

**Iron overload**	T2* (ms)
normal	>20
iron overload	<20
severe iron overload	<10

## Regional Measurements and Cardiac Strain

### CMR acquisition parameters

A number of imaging methods have been developed to acquire cardiac strain information from CMR including cine CMR, tagged MR, phase-contrast CMR (PC-CMR), velocity encoded CMR, displacement encoding with stimulated echoes (DENSE), and strain-encoding (SENC) [88,89]. However, tagged CMR remains a widely validated reproducible tool for strain estimation. The method is used in clinical studies and is considered the reference standard for assessing regional function [90,91].

### CMR analysis methods

Cardiac strain is a dimensionless measurement of the deformation that occurs in the myocardium. Cardiac strain can be reported as three normal strains (circumferential, radial, and longitudinal) and six shear strains—the angular change between two originally mutually orthogonal line elements, with the more clinically investigated shear strain in the circumferential-longitudinal shear strain (also known as torsion).

There are a number of different methods to quantify strain: registration methods, feature-based tracking methods, deformable models, Gabor Filter Banks, optic flow methods, harmonic phase analysis (HARP) [92], and local sine wave modeling (SinMod) [88]. Technical review papers for these methods can be found in the following literature [93-96]. HARP has become one of the most widely used methods for analyzing tagged MR images for cardiac strain, in part due to its large scale use in the Multi-Ethnic Study of Atherosclerosis (MESA) trial [92,97].

Strain patterns are reported according to the 16 and 17 segment model of the American College of Echocardiology. Consistent manual tracing of the endocardial and epicardial contours is necessary to reproducible strain results. With tagged CMR, midwall strain is preferred to epicardial and endocardial strain to maximize the amount of tagging data available for strain calculations [95,98]. With HARP analysis such as that used in the MESA trial [92], careful selection of the first harmonic is necessary.

### Demographic parameters

With tagged CMR, it has been noted that age is associated with decrease in peak circumferential or longitudinal shortening [99,100]. In tagged CMR studies, gender also affects normal values. Cardiac strain values for women are higher than those of men [101,102].

### Studies included in this review

Several studies have presented cohorts of normal individuals for determining normal strain of the left ventricle. For the purpose of this review, only cohorts of 30 or more normal subjects using SPAMM tagging have been included. (Feature tracking methods are being developed for strain, but are being validation in comparison to SPAMM tagging.) Inclusion criteria include a full description of the subject cohort (including the analysis methods used), age and gender of subjects. Table 46 represents a summary of publications reporting normal values for midwall strain that fit the criteria [89,90,98].

**Table 46:** **Normal values for circumferential, longitudinal und radial strain (maximal strain in %; mean±SD) according to previous publications**

**Author**	**n**	**Age (yrs)**	**Acquis. method**	**Estimation method**	**Tagged resol.**	**FS (T)**	**base**	**mid**	**ap**	**sept**	**ant**	**lat**	**inf**
**Circumferential Strain**													
Moore (2000) [98]	31	37 ± 11	SPAMM	FindTags	6 mm	1.5	−0.19±0.04	−0.19±0.05	−0.22±0.05	−0.17±0.03	−0.22±0.05	−0.22±0.04	−0.18±0.05
Cupps (2010) [89]	50	32.8±10.6	SPAMM	finite element model	7 mm	1.5	−0.19±0.04	−0.21±0.04	−0.20±0.04	−0.18±0.03	−0.20±0.04	−0.23±0.04	−0.19±0.04
Del-Canto (2013) [90]	36	58.8 ± 11.6	SPAMM	SinMod	7 mm	1.5	−0.17 ± 0.03	−0.20 ± 0.03	−0.20 ± 0.03	−0.16 ± 0.04	−0.20 ± 0.04	−0.22 ±0.04	−0.18 ± 0.04
Venkatesh (2014) [103]	129	58.8 ± 9.3	SPAMM	HARP	7 mm	1.5	−15.1 ± 3.2	−18.0 ± 2.2	−17.9 ± 2.5				
**Longitudinal Strain**													
Moore (2000) [98]	31	37 ± 11	SPAMM	FindTags	6 mm	1.5	−0.15±0.03	−0.15±0.03	−0.19±0.04	−0.16±0.04	−0.16±0.04	−0.16±0.04	−0.16±0.04
Cupps (2010) [89]	50	32.8±10.6	SPAMM	finite element model	7 mm	1.5	−0.13±0.04	−0.15±0.03	−0.18±0.05	−0.16±0.04	0.15±0.04	−0.15±0.04	−0.15±0.05
**Radial Strain**													
Moore (2000) [98]	31	37 ± 11	SPAMM	FindTags	6 mm	1.5	0.45±0.26	0.42±0.26	0.48±0.33	0.41±0.19	0.54±0.28	0.46±0.23	0.38±0.38
Del-Canto (2013) [90]	36	58.8 ± 11.6	SPAMM	SinMod	7 mm	1.5	0.13 ± 0.04	0.09 ± 0.04	0.12 ± 0.04	0.09 ±0.05	0.10 ±0.05	0.12 ±0.07	0.14±0.07
Venkatesh (2014) [103]	129	58.8 ± 9.3	SPAMM	HARP	7 mm	1.5	27.4 ± 5.6	24.8 ± 5.3	23.4 ± 6.7				

yrs = years; Acquis. = Acquisition; resol. = resolution; FS = field strength; T = Tesla; SPAMM = spatial modulation of the magnetization; mid = mid cavity; ap = apical level; sept = septal; ant = anterior; lat = lateral; inf = inferior.

With tagged CMR, normal midwall circumferential [89,92,93,98,101,104] and longitudinal [89,96,98,104] strain values are relatively comparable between studies. For radiation strain, moderate differences between published results exist for reference values, probably due to low tag density by most methods in the radial direction [90,96-98,101,104].

## Conclusions

Cardiovascular magnetic resonance enables quantification of various functional and morphological parameters of the cardiovascular system. This review lists reference values and their influencing factors of these parameters based on current CMR techniques and sequences.

Advantages of a quantitative evaluation are a better differentiation between pathology and normal conditions, grading of pathologies, monitoring changes under therapy, and evaluating prognosis and the possibility of comparing different groups of patients and normal subjects.

## Abbreviations

CMR: Cardiovascular magnetic resonance
SSFP: Steady-state free precession
LV: Left ventricle
Yrs: Years
FGRE: Fast gradient echo
EDV: End-diastolic volume
BSA: Body surface area
ESV: End-systolic volume
SV: Stroke volume
EF: Ejection fraction
SD: Standard deviation
SD_p_: Pooled standard deviation
Mean_p_: Pooled weighted mean
RV: Right ventricle
PFRE: Peak filling rate early
PFRA: Peak filling rate active
AVPD: Atrioventricular plane descent
3D: 3-dimensional
CI: Confidence interval
RA: Right atrium
LA: Left atrium
AP: Anteroposterior
4ch: 4-chamber view
2ch: 2-chamber view
3ch: 3-chamber view
Max.: Maximal
Min.: Minimal
CI: Cardiac index
LVMT: Left ventricular myocardial thickness
SVC: Superior vena cava
IVC: Inferior vena cava
Vol.: Volume
PC: Phase contrast
4D: 4-dimensional
V_enc_: Flow encoding velocity
AHA: American Heart Association
MRA: Magnetic resonance angiography
MDT: Mitral deceleration time
E/A ratio: Ratio of the mitral early (E) and atrial (A) components of the mitral inflow velocity profile
CT: Computed tomography
BMI: Body mass index
Prox.: Proximal
Decs.: Descending
AS: Aortic sinus
STJ: Sinotubular junction
AA: Ascending aorta
BCA: Proximal to the origin of the brachiocephalic artery
IR: Isthmic region
DA: Descending aorta
PWV: Pulse wave velocity
ECV: Extracellular volume
MOLLI: Modified Look-Locker Inversion Recovery
ShMOLLI: Shortened Modified Look-Locker Inversion Recovery
SASHA: Saturation Recovery Single-Shot Acquisition
EQ-CMR: Equilibrium cardiovascular magnetic resonance
FLASH: Fast low angle shot
G(R)E: Gradient echo
BH: Breath hold
VAST: Variable sampling of k-space
LL: Look-Locker
Hb: Heart beat
SAX: Short axis
DENSE: Displacement encoding with stimulated echoes
SENC: Strain encoding
HARP: Harmonic phase analysis
SinMod: Sine wave modeling
MESA: Multi-Ethinic Study of Atherosclerosis
Resol: Resolution
SPAMM: Spatial modulation of the magnetization
mid: Mid cavity
ap: Apical level
sept: Septal
ant: Anterior
lat: Lateral
inf: Inferior
FS: Field strength
